# Mechanical Contact Conditions in Wearable Reflectance Photoplethysmography: Scoping Review

**DOI:** 10.2196/99333

**Published:** 2026-08-24

**Authors:** Chenxi Yang, Jiahang Xie, Zifei He, Jianqing Li, Chengyu Liu

**Affiliations:** 1 School of Instrument Science and Engineering Southeast University Nanjing, Jiangsu China; 2 State Key Laboratory of Digital Medical Engineering Southeast University Nanjing, Jiangsu China

**Keywords:** contact force, contact pressure, wearable photoplethysmography, reflectance photoplethysmography, signal quality, waveform morphology, artifact susceptibility, temporal stability, physiological parameter estimation

## Abstract

**Background:**

Cardiovascular diseases remain a major global health burden, highlighting the need for long-term physiological monitoring. Photoplethysmography (PPG) is widely used in wearable devices for noninvasive monitoring of heart rate (HR), rhythm, and oxygen saturation in mobile health (mHealth) apps. However, the reliability of wearable reflectance PPG depends on sensing conditions, including sensor-skin contact force and pressure.

**Objective:**

This scoping review maps how contact force and contact pressure have been defined, controlled, measured, represented, and reported in wearable or wearable-relevant reflectance PPG studies; characterizes the reported signal-, waveform-, feature-, and task-level responses under different contact conditions; and identifies methodological and evidence gaps.

**Methods:**

A comprehensive literature search was conducted in PubMed, IEEE Xplore, Scopus (Elsevier), and Web of Science Core Collection (Clarivate) from database inception to June 1, 2026. Studies were eligible if they addressed contact force or contact pressure in wearable or wearable-relevant reflectance PPG and reported measurement approaches, measurement sites and device configurations, force or pressure representation, or PPG responses across signal quality, waveform and feature characteristics, and downstream physiological estimation. Database filters were applied, where available, to restrict results to English-language publications. Search results were imported into EndNote for deduplication. Database searches were supplemented by reference-list screening. After screening, 53 reports were sought for retrieval; 1 was not retrieved, 52 were assessed at full text, and 21 studies met the inclusion criteria.

**Results:**

The 21 included studies showed substantial heterogeneity, with sample sizes ranging from single-participant experiments to a wrist PPG dataset including 1142 participants. Most human studies recruited healthy volunteers, whereas some used public datasets or tissue-vessel phantoms, and 1 combined theoretical modeling with human-participant validation. The mapped evidence identified contact force and contact pressure as important measurement conditions in wearable reflectance PPG. Across the included studies, different contact conditions were associated with changes in alternating current/direct current components, amplitude- and morphology-related features, derivative-based indices, wavelength-dependent responses, and fiducial-point detection. Several studies also examined downstream physiological tasks, including HR, oxygen saturation, pulse transit or arrival time, blood pressure–related estimates, and HR variability.

**Conclusions:**

The evidence mapped in this scoping review supports a multilevel conceptual pathway in wearable reflectance PPG, in which mechanical conditions at the sensor-skin interface are associated with changes in PPG signal characteristics, derived features, and, in a smaller body of studies, downstream physiological estimation. Evidence remains limited by small samples, short-term controlled protocols, and inconsistent reporting of mechanical parameters, including units, contact area, and probe geometry. Insufficient population diversity and limited free-living validation further restrict generalizability. Future research should standardize reporting of contact conditions and device geometry, incorporate real-world validation, and develop force-aware signal-quality assessment algorithms, adaptive attachment designs, and context-aware models to improve mHealth and cardiovascular monitoring.

## Introduction

### Background

Cardiovascular diseases remain a major cause of morbidity, mortality, and health care burden worldwide [1,2]. As many cardiovascular conditions may remain asymptomatic in their early stages, timely risk identification and intervention are critical for improving outcomes and preventing disease progression [3,4]. However, current screening and diagnostic approaches still largely depend on professional medical settings and centralized equipment, limiting their accessibility for long-term follow-up and routine management. Therefore, scalable and low-cost digital health technologies for continuous monitoring are increasingly important in cardiovascular disease prevention and management [5]. In this context, photoplethysmography (PPG) has become one of the most widely used sensing modalities in digital health and mobile health (mHealth) apps because of its noninvasive nature, convenience, and suitability for long-term use.

By optically detecting pulsatile blood volume changes, PPG can support the assessment of heart rate (HR), oxygen saturation, blood perfusion, and certain vascular function–related indices [6]. With advances in sensors, wearable devices, and artificial intelligence [7], PPG has expanded from traditional pulse and oxygen saturation monitoring to broader applications such as HR monitoring [8], blood pressure (BP) assessment, oxygen saturation estimation [9], and cardiovascular health monitoring. With the rapid development of wearable technologies, PPG has been widely integrated into smartwatches, wristbands, patches, and other devices for continuous and noninvasive health monitoring [10-12]. In homes, communities, and other nonclinical settings, these wearable PPG systems offer practical advantages for remote monitoring and proactive health management, highlighting their strong potential for large-scale cardiovascular health assessment [5].

At the same time, the effectiveness of PPG in wearable applications is fundamentally constrained by signal quality, which in turn is highly dependent on specific sensing conditions, including sensor geometry, the skin-sensor interface, ambient light, contact force, and measurement-site characteristics [13,14]. Previous studies have identified contact force and contact pressure as relevant interface conditions associated with differences in sensor-skin coupling, local vascular loading, waveform quality, and downstream interpretability [13,15]. However, contact force has often been treated as a secondary issue. In practice, sensor-skin pressure is not constant and changes with wearing conditions. Too little contact force weakens mechanical coupling, facilitates relative motion, and increases susceptibility to ambient light and random noise. Excessive contact force can compress superficial vessels and capillary beds, alter local microcirculation, suppress pulsatile blood-volume changes, and distort the waveform [16]. Across analytical levels, contact conditions have been reported in relation to differences in alternating current/direct current (AC/DC) components, waveform morphology, signal-quality indices, and feature stability [16,17]. In wearable settings, these differences have also been examined alongside posture, motion, tissue deformation, strap fit, and downstream tasks, including peripheral oxygen saturation (SpO_2_) estimation [15,18,19], heart-rate monitoring, heart rate variability (HRV) analysis, pulse arrival time (PAT)/pulse transit time (PTT) extraction, blood pressure modeling, and other PPG-driven applications [15,20,21].

### Related Reviews

To position this scoping review within the existing literature, we reviewed representative PPG-related reviews published over the past decade. Existing reviews have addressed PPG from several perspectives, including its physiological basis, waveform characteristics, signal processing methods, and applications in HR, BP, oxygen saturation, and arrhythmia detection [7-10,22-29]. Other reviews have focused on wearable PPG devices, summarizing sensor structures, measurement sites, device forms, system architectures, and application scenarios [30-34]. In addition, some reviews have discussed PPG signal analysis from the perspectives of signal quality, artifact sources, dataset resources, and algorithmic tools [7,29,35-37]. Overall, these reviews have mainly approached PPG from the perspectives of signal technology, wearable implementation, signal quality, or downstream physiological applications. Existing reviews have acknowledged contact pressure, wearing tightness, and sensor-skin interface stability as factors related to PPG measurement quality [29-32], but these issues have generally been treated as secondary acquisition considerations within broader discussions of wearable devices, signal quality, or physiological applications. As a result, limited attention has been given to how contact force and contact pressure are defined, measured, controlled, represented, and reported across wearable reflectance PPG studies. This scoping review addresses this gap by using contact force and contact pressure as the central organizing concepts and mapping measurement and control approaches, anatomical sites, device configurations, reporting practices, and corresponding PPG responses across signal quality, waveform characteristics, derived features, and downstream physiological estimation. It further examines the distribution of evidence across populations, experimental contexts, and protocol durations to clarify the methodological structure of the field and identify areas in which evidence remains limited.

### Objectives

This scoping review aimed to map and characterize the existing literature on contact force and contact pressure in wearable reflectance PPG. The review was guided by the following research questions: (1) How have contact force and contact pressure been defined, applied, controlled, measured, estimated, and reported in wearable reflectance PPG studies? (2) Which signal-level, waveform-level, feature-level, signal-quality, and task-level outcomes have been investigated under different contact conditions? (3) How is the available evidence distributed across measurement sites, sensor configurations, study populations, experimental settings, and outcome categories? In addition, this review aimed to identify current evidence gaps and methodological challenges to inform future force-aware wearable PPG acquisition, sensor-skin interface design, algorithm development, and standardization in wearable cardiovascular monitoring.

## Methods

### Protocol and Registration

No review protocol was registered or publicly published for this scoping review. The eligibility criteria, information sources, search strategy, study-selection procedures, and initial data-charting framework were established before full-text screening. The data-charting categories were subsequently refined iteratively during data extraction as familiarity with the terminology, methodological diversity, and outcome categories of the included studies increased. The final review methods are reported in this section and Multimedia Appendices 1-3.

### Study Design

This study used a scoping review methodology to address the breadth and heterogeneity of the available evidence on contact force and contact pressure in wearable reflectance PPG. The review mapped how these contact conditions were defined, measured, and reported, together with the associated PPG outcomes and evidence gaps. The review was conducted following the framework proposed by Arksey and O’Malley [38] and was reported in accordance with the PRISMA-ScR (Preferred Reporting Items for Systematic Reviews and Meta-Analyses extension for Scoping Reviews) checklist (Multimedia Appendix 1) [39]. In addition, the literature search was reported according to the PRISMA-S (Preferred Reporting Items for Systematic Reviews and Meta-Analyses literature search extension; Multimedia Appendix 2) [40] checklist to strengthen the transparency and reproducibility of the search process.

### Eligibility Criteria

#### Inclusion Criteria

Peer-reviewed journal articles and conference proceedings published in English were considered for inclusion. Eligible studies were required to examine or report contact force, contact pressure, or related sensor-skin interface conditions in wearable or wearable-relevant reflectance PPG and to provide relevant evidence on methodological characteristics, including measurement sites and device configurations, and PPG outcomes across signal-quality, waveform, feature, artifact-susceptibility, and downstream-task domains. Specifically, studies were required to meet all 3 core criteria: (1) the study must involve contact force, contact pressure, or sensor-skin interface conditions (including direct measurement, controlled application, or qualitative description); (2) the study must use wearable or wearable-relevant reflectance-mode PPG systems; and (3) the study must report at least one PPG-related methodological characteristic or outcome that was observed under, compared across, or interpreted in relation to the contact condition. In this review, contact force refers to the total mechanical load applied at the sensor-skin interface, whereas contact pressure refers to the distribution of that load over the effective contact area. Terms and units were retained as reported by the source studies and were not treated as interchangeable unless sufficient contact-area or geometric information was available.

#### Exclusion Criteria

Non–peer-reviewed publications, non-English articles, and studies without accessible full text were excluded. Studies were further excluded if they met 1 or more of the following criteria: (1) did not address contact force, contact pressure, or sensor-skin interface–related factors in the context of PPG signal acquisition or analysis; (2) focused solely on transmission-mode PPG systems without clear relevance to wearable or wearable-relevant applications; or (3) were review articles, editorials, commentaries, conference abstracts, or other nonoriginal research publications.

### Information Sources and Search

To identify relevant studies, a comprehensive literature search strategy was designed by the review authors and further refined through iterative discussion. The search strategy was developed with reference to PRISMA-S reporting principles and methodological recommendations from Cochrane and Joanna Briggs Institute, emphasizing the combined use of controlled vocabulary terms, free-text terms, field modifiers, and a broad range of synonyms. The search strategy was built around 3 concepts: PPG and wearable reflectance PPG; contact force/contact pressure and the sensor-skin interface; and signal quality, waveform morphology, feature extraction, and downstream physiological estimation. The strategy combined controlled vocabulary terms, where available, with free-text keywords and was first developed for PubMed before being translated to the other databases with syntax-specific adaptations. The search was conducted in PubMed, IEEE Xplore, Scopus (Elsevier), and Web of Science Core Collection (Clarivate), accessed through the university library, covering records from database inception to June 1, 2026. Search terms included concepts related to PPG, wearable-device applications, contact conditions, and relevant signal- and task-level outcomes. Database filters were applied, where available, to restrict results to English-language publications. Review articles, editorials, commentaries, conference abstracts, and other nonoriginal publications were excluded during screening because this scoping review aimed to map original research evidence. The complete database-specific search strategies are reported in Multimedia Appendix 3. All search results were exported as RIS files and imported into EndNote (Clarivate Analytics) for reference management and deduplication. Duplicate records were first identified using EndNote’s automated duplicate-detection function and then checked manually. The database searches were supplemented by backward citation searching of the reference lists of included studies. Potentially relevant reports were cross-checked against the original database exports using DOI and title information. No formal peer review of the search strategy by an information specialist or librarian was performed; however, the search strategy was iteratively reviewed by the author team and checked against PRISMA-S reporting items.

### Selection of Sources of Evidence

All retrieved records were imported into EndNote for reference management, and duplicate records were removed before screening. Title and abstract screening was independently conducted by 2 reviewers (JX and ZH) to identify potentially relevant studies. Each record was assessed by both reviewers, and studies deemed relevant were subsequently subjected to full-text review. Full-text articles were independently evaluated by the same 2 reviewers according to the predefined inclusion and exclusion criteria. Any disagreements arising during the title and abstract screening or full-text review stages were resolved through discussion; if consensus could not be reached, a third reviewer (CY) was consulted for final adjudication. The reference-list entries identified through citation searching were screened by the same reviewers, and potentially relevant reports were assessed using the same inclusion and exclusion criteria applied to reports identified through the database searches. Reports were assigned to the citation-searching pathway only after confirming that they were not represented in the original database exports. Given the scoping nature of this review, the selection process aimed to map the available evidence broadly and transparently according to the predefined eligibility criteria.

### Data Charting Process and Data Items

Relevant data were extracted from the included studies using a standardized data-charting approach. Extracted items covered study and publication characteristics; participant or experimental-platform characteristics; measurement sites and device configurations; contact-condition terminology, measurement or control approaches, and force or pressure representation; experimental protocols; key methodological reporting details; and reported PPG outcomes and main findings across signal and signal-quality, waveform, feature, artifact-susceptibility, and downstream-task domains. The data-charting process was iterative, allowing refinement of categories as familiarity with the literature increased. Information unavailable in the source articles was recorded as not reported.

### Critical Appraisal of Individual Sources of Evidence

A formal critical appraisal of individual sources of evidence was not conducted because this scoping review aimed to map the breadth, characteristics, and methodological heterogeneity of the available evidence rather than to assess the certainty of the evidence or generate pooled effect estimates.

### Synthesis of Results

To address the review questions, the charted data were first summarized using descriptive numerical analysis. Frequencies and percentages were calculated for key study, methodological, and outcome characteristics, with categories treated as nonmutually exclusive where appropriate. The individual sources of evidence were subsequently analyzed and synthesized narratively to compare methodological approaches, study contexts, and reported PPG outcomes across contact conditions. Finally, cross-study synthesis was conducted using evidence mapping, study-level linkage analysis, and study-level methodological and analytical coverage mapping to characterize the distribution, connections, and breadth of the available evidence. Owing to substantial heterogeneity in study designs, contact-condition definitions, measurement protocols, and outcome measures, a meta-analysis was not conducted.

## Results

### Selection of Sources of Evidence

**Figure 1:**
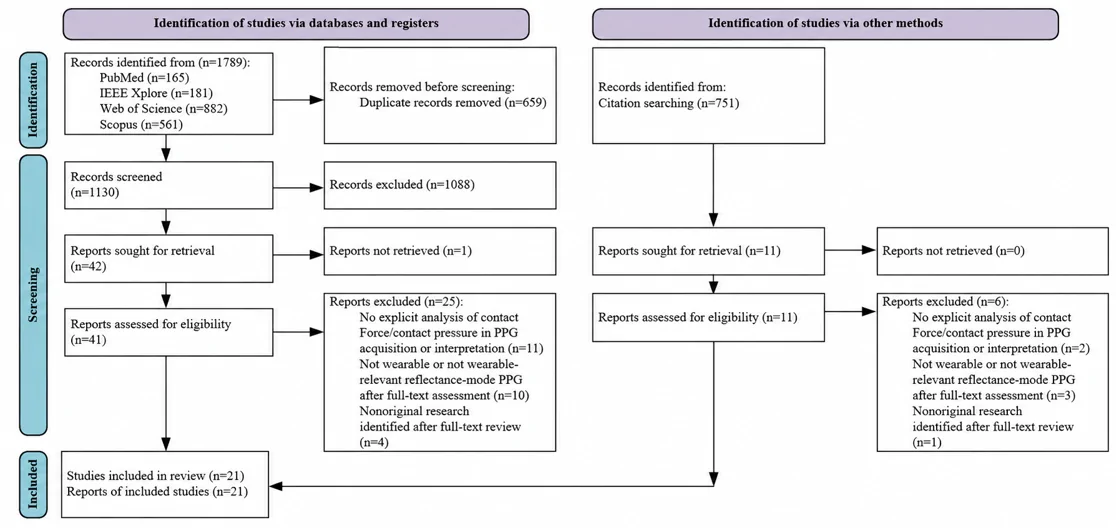
PRISMA (Preferred Reporting Items for Systematic Reviews and Meta-Analyses) flow diagram. PPG: photoplethysmography.

Figure 1 presents the PRISMA (Preferred Reporting Items for Systematic Reviews and Meta-Analyses) flow diagram illustrating the identification, screening, eligibility assessment, and final inclusion of studies. Database searches identified 1789 records, of which 1130 remained after deduplication and were screened; 16 reports were included through the database pathway. In addition, 751 reference-list entries were screened through citation searching, resulting in 5 additional included reports. Overall, 21 studies were included in the review.

### Characteristics of Sources of Evidence

#### Characteristics of Publication and Baseline

Multimedia Appendix 4 summarizes the baseline characteristics of the 21 included studies, including publication year, publication type, country or region, and primary study focus [15,16,18,20,21,41-56]. The included studies were published between 2006 and 2026. Of these 21 studies, 15 (71%) [15,16,20,21,42-52] were published from 2020 onward, showing that most of the included evidence was published in recent years; besides, 16 (76%) [15,16,21,41-44,46-51,53-55] were journal articles and 5 (23.8%) [18,20,45,52,56] were peer-reviewed conference papers. In terms of country or region, the included studies were mainly from the United States (6/21, 29%) [18,42,47,48,52,56], China, including Hong Kong (3/21, 14%) [15,43,54], the United Kingdom (2/21, 10%) [16,46], Finland (2/21, 10%) [20,51], and Italy (2/21, 10%) [21,49], with 1 study each from South Korea [41], Slovakia [44], Canada [45], Spain [50], Latvia [53], and Israel [55]. Country or region was summarized according to the reported study location when available; when the study location was not explicitly stated, it was assigned according to the first or corresponding author’s institutional affiliation.

#### Characteristics of Study Designs and Research Focus

The study-level characteristics of all included sources are summarized in Multimedia Appendix 4, with additional methodological and outcome details provided in Tables 1-3 and Multimedia Appendix 5. The included studies contributed to 3 broad and nonmutually exclusive research domains: contact-condition measurement, control, sensing, or estimation; directly measured signal-, waveform-, or feature-level responses; and directly evaluated downstream wearable monitoring tasks. Of the 21 included studies, 4 (19%) [41,43-45] had a primary methodological focus, 16 (76%) [15,16,18,20,41-45,47,49-51,53,55,56] reported at least 1 directly measured signal-, waveform-, or feature-level response, and 10 (48%) [15,18,21,42,47,49,51,52,54,56] directly evaluated at least 1 downstream task under different contact conditions. As these domains were nonmutually exclusive, individual studies could contribute to more than 1 domain.

**Table 1:** Summary of contact-force control and sensing methods.

Study	Study population and sample size	Measurement site/experimental condition	Method type	How force is controlled	How force is sensed	Force/pressure reporting and convertibility	Key hardware/implementation	Main information provided
Sim et al [41]	Healthy human-participant validation Main posture-change experiment: 1 healthy participant Additional contact-force–dependency experiment: 4 participants. Male/female distribution: not reported	Radial artery at the wrist	Active closed-loop control	Probe displacement is actively adjusted with a thermo-pneumatic actuator to maintain constant contact force in real time.	A FlexiForce A201 flexible force sensor measures force through force-dependent conductance changes.	Contact force Unit: Newton; target force: 0.6 N; measurable range: 0.2-4.0 N Effective contact area: not reported Contact-force–related signal Unit/range: not reported	Wrist platform comprising a force regulator, main body, force sensor, and reflectance PPG^a^ probe.	Demonstrates a closed-loop contact-force regulation strategy for improving wrist PPG stability under posture-related contact changes.
Liu et al [43]	Sample size and participant health status: not reported	Wrist; elastic wristband positioned over the radial and ulnar artery regions.	Multipoint synchronous monitoring	More of a force-sensing/regulation platform than a strict actuator-based closed loop; emphasis is on synchronous spatial monitoring.	Four ceramic piezoelectric sensors track changes in mechanical stress/contact force.	Geometry/contact area: wrist-contact PPG and piezoelectric sensors; sensor diameter reported as 12 mm, but the effective skin-contact area for pressure conversion was not reported. Contact force; authors used the term contact pressure force. Unit/range: Newton; 5 force levels	Wearable platform integrating 9 PPG sensors and 4 piezoelectric sensors. The PPG sensors form a 3 × 3 array embedded in an elastic wristband, enabling simultaneous acquisition of 9 PPG channels and 4 contact force channels.	Provides a multipoint view of how local contact-force variation and spatial sensor position affect wrist PPG waveform behavior.
Přibil et al [44]	6 healthy volunteers: 4 males and 2 females Mean age 56 (SD 8) years All healthy volunteers	Left and right index fingers; five force levels	Direct contact force	No active control is emphasized; the main goal is direct quantification of probe-skin force.	A force-sensitive resistor directly measures probe-skin contact force.	Male: 0.147-1.078 N; female: 0.147-0.753 N Geometry/area: The force-sensitive resistor active sensing region had a diameter of 4 mm, but the effective skin-contact area was not reported; pressure in mm Hg (millimeters of mercury)/kPa (kilopascals) was not reported.	PPG probe integrated with humidity, temperature, and force-sensitive resistor sensing.	Provides direct sensor-level documentation of finger PPG changes under different applied contact-pressure-force levels.
Fortin et al [45]	1 participant: male (28 years old), right-handed, pale White skin tone; self-administered protocol because of COVID-19 self-isolation restrictions.	Wrist and index fingertip	Indirect force estimation	No active force-control device is added; force is changed experimentally using a clamp and adjustment screw.	A miniature load cell provides the ground-truth force during development; the long-term aim is to estimate force from raw PPG alone.	Contact force Unit/range: Newton; 0-2.6 N in 0.2 N increments Effective contact area: not reported	3D-printed clamp, 500 g load cell, HX711 module, and Arduino. Force sampled from 0 to 2.6 N in 0.2 N steps. 55 features extracted from raw PPG and modeled with a bagged-tree approach.	Shows the feasibility of estimating contact force from raw PPG features without adding a dedicated force sensor to the final wearable system.

^a^PPG: photoplethysmography.

**Table 2:** Measurement sites and wearable configurations in representative studies.

Study	Study design	Sample size and participant characteristics	Main measurement site	Wearable configuration/device form	Reference/auxiliary channel	Main findings
Charlton et al [46]	Measurements in different postures and sensor heights to examine effects of posture and sensor position relative to the heart on wrist PPG^a^ signal quality.	1142 participants from the Aurora-BP dataset with varying ages and health statuses.	Wrist	Wrist-worn reflectance PPG	Posture and sensor height considered	Wrist PPG signal quality was strongly shaped by body posture and sensor height relative to the heart, indicating that wrist-site performance depends on acquisition geometry rather than anatomical site alone.
Ho et al [47]	A wrist-finger dual-channel dataset study for contact-pressure effects on wrist PPG morphology.	27 healthy young participants (16 males and 11 females)	Wrist	Force-adjustable wrist module	High-perfusion fingertip reference	Wrist posture changed sensor-skin contact pressure and produced different wrist PPG morphologies; the wrist setup supports analysis of wrist-site distortion in smartwatch-like monitoring
Dresher and Mendelson [18]	A forehead reflectance pulse-oximetry walking study under different contact pressures; involved an oxygen-saturation monitoring context	10 healthy volunteers; male/female distribution: not reported	Forehead above the eye	Elastic-headband–based forehead reflectance pulse oximeter with custom housing	Fingertip reference	The forehead above the eye was evaluated as a motion-tolerant reflectance pulse-oximetry site, but its performance depended on headband fixation and appropriate sensor-skin pressure during walking.
Chan et al [48]	A wearable reflectance pulse-oximetry study at the sternum; involved a continuous SpO_2_^b^ monitoring context	Total sample size, sex distribution, and participant characteristics: not reported	Mid-sternum	Chest/central reflectance PPG	Continuous SpO_2_ task	The mid-sternum was assessed as a central wearable reflectance PPG site using a chest patch; this configuration enabled continuous SpO_2_ monitoring but required attention to weak local perfusion and respiratory artifacts.

^a^PPG: photoplethysmography.

^b^SpO_2_: peripheral oxygen saturation.

**Table 3:** Static and dynamic study protocols in representative contact-force research.

Study	Study design/protocol characteristics	Sample size and participant characteristics/platform	Measurement site	Protocol design	Force/pressure reporting	Main observation and finding
Scardulla et al [49]	A dynamic in vivo step-exercise wrist PPG^a^ study evaluating how preset wristband contact pressure affects PPG-derived heart-rate accuracy under different stepping intensities.	17 participants (12 males and 5 females), with a mean age of 36 (SD 11) years; health status was not explicitly reported.	Wrist reflectance PPG with electrocardiogram chest-strap reference.	Participants stepped on a 22.5 cm platform at 90, 120, and 140 bpm for 60 seconds, preceded by 10 seconds of rest. Three contact pressures were randomized across 3 activity intensities, yielding 9 tests per participant.	Contact pressure Unit/range: mm Hg (millimeters of mercury); 12, 33, and 54 mm Hg. Geometry/contact area: contact area = 473 mm2	Dynamic step-exercise protocol: PPG-derived heart rate was compared with electrocardiogram-derived heart rate under 3 contact pressures and 3 stepping intensities. Contact pressure affected heart rate agreement during movement, showing that pressure optimization remains important in dynamic wrist PPG recordings.
Sirkiä et al [20]	A quasi-static in vivo finger multiwavelength PPG pressure-ramp study examining how controlled external finger pressure affects wavelength-dependent PPG waveforms, vascular occlusion/opening behavior, and pulse-foot timing.	3 volunteers (1 female); health status and age were not reported	Index finger of the right hand; 5-wavelength multiwavelength PPG with controlled external pressure.	External pressure was continuously increased by lowering a bar with a stepper motor until the maximum allowed pressure was above systolic blood pressure and then released. Multiwavelength PPG and pressure signals were recorded synchronously, with 3 measurements per volunteer and reference blood pressure after each measurement.	External pressure/contact pressure. Unit/range: mm Hg; pressure was increased above systolic blood pressure and then released. Geometry/contact area: multiwavelength PPG sensor printed circuit board diameter of approximately 9.6 mm; effective skin-contact area not reported.	Quasi-static finger pressure-ramp protocol: multiwavelength PPG was observed during external pressure increase and release. Pressure produced wavelength-dependent blockage/opening behavior and pulse-timing changes, indicating that external pressure alters vascular responses differently across optical wavelengths.
Scardulla et al [21]	A standardized dynamic in vivo treadmill wrist PPG study evaluating heart-rate accuracy, precision, and uncertainty under different strap pressures and exercise speeds.	25 healthy volunteers; male/female distribution was not reported.	Wrist PPG prototype with electrocardiogram chest-strap reference.	For each pressure condition, blood pressure was measured first. Participants then performed treadmill trials at 3, 6, and 8 km/h for 90 seconds each with 60-second rest intervals, followed by a 5-minute seated PPG recording and another blood pressure measurement. The full sequence was repeated at 20, 60, and 75 mm Hg.	Contact pressure. Unit/range: mm Hg; 20, 60, and 75 mm Hg. Effective contact area: not reported.	Dynamic treadmill protocol: heart rate accuracy, precision, and uncertainty were evaluated under 3 strap pressures and 3 treadmill speeds. Intermediate pressure, especially around 60 mm Hg, generally provided better PPG-heart rate performance than lower or higher pressures.
May et al [16]	A static in vitro tissue-vessel phantom study examining how progressively increased sensor contact pressure affects reflectance PPG signal quality and morphological features under simulated blood pressure states.	In vitro tissue-vessel phantom; no human participants; sex distribution not applicable.	Above the embedded vessel in the phantom.	Under pulsatile flow fixed at 60 bpm, 4 hemodynamic states were simulated: hypotension, normotension, stage 1 hypertension, and stage 2 hypertension. A linear actuator progressively increased sensor pressure until complete vessel occlusion.	Contact pressure. Unit/range: mm Hg; continuously increased until complete vessel occlusion; optimal range was reported as 35.1-48.1 mm Hg. Effective contact area: not reported.	Static phantom protocol: signal-to-noise ratio and 17 morphological features were observed as contact pressure increased until vessel occlusion. PPG signal quality and morphology changed nonlinearly with pressure, with an optimal pressure range before complete occlusion.
Ho et al [47]	A static/quasi-static seated wrist-finger dual-channel PPG dataset study examining how wrist contact-pressure variation affects wrist PPG morphology, with fingertip PPG recorded simultaneously as a reference.	27 healthy young participants (16 males and 11 females); mean age 24.3 (SD 2.74) years	Wrist PPG with fingertip PPG reference	Each participant completed 6 sessions. Each session included blood pressure measurement, contact-pressure adjustment, and 4 minutes of seated recording, with a 2-minute rest between sessions. Wrist PPG, fingertip PPG, load cell, electrocardiogram, blood pressure, and SpO_2_^2^ were synchronized.	Contact pressure. Unit/range: load-cell reading; gradually increased across 6 sessions. Geometry/contact area: load cell placed between the wrist PPG sensor and movable jaw; effective skin-contact area was not reported.	Static/quasi-static seated wrist-finger protocol: wrist PPG morphology was observed across 6 contact-pressure sessions with fingertip PPG as a reference. Wrist contact-pressure variation systematically changed wrist PPG morphology, supporting dual-site recordings to characterize pressure-related wrist-site distortion.

^a^PPG: photoplethysmography.

^b^SpO_2_: peripheral oxygen saturation.

#### Characteristics of Participants and Data Sources

The included sources of evidence varied substantially in terms of participant characteristics and data sources. Most human-participant studies involved healthy volunteers [18,20,21,41,42,44,45,47,49-53,56], whereas some studies used larger datasets or study populations with broader participant characteristics [46,52]. Sample sizes ranged from single-case feasibility studies [41,45] to large-scale wrist PPG datasets [46]. Several studies did not fully report participant age, sex distribution, or health status [18,43,48,50,55], limiting characterization of population diversity across the evidence base. In addition to the in vivo human studies, the evidence base included an in vitro tissue-vessel phantom study [16] and a study combining theoretical modeling with human-participant experimental validation [54].

#### Characteristics of Measurement Sites and Wearable Reflectance PPG Configurations

Measurement-site categories were nonmutually exclusive because some studies included more than 1 PPG acquisition site or used a secondary PPG site as a reference channel. Of the 21 included studies, 10 (48%) [15,21,41,43,45-47,49,52,55] involved wrist-based configurations, 9 (43%) [20,42,44,45,47,50,51,54,55] involved finger-based measurements, and 3 (14%) [18,55,56] involved forehead-based configurations. One study (5%) [48] examined a sternum-based configuration, 1 (5%) [53] examined other anatomical sites, and 1 (5%) [16] used an in vitro tissue-vessel phantom rather than a human anatomical measurement site. As several studies involved more than 1 measurement site, the summed percentages exceeded 100%. Primary device or experimental configurations were coded into mutually exclusive categories according to the principal PPG setup used in each study. Of the 21 included studies, 7 (33%) [15,21,41,43,46,49,52] used single-site wrist-based systems, 6 (29%) [20,42,44,50,51,54] used single-site finger-based systems, and 3 (14%) [45,47,55] used multisite configurations involving more than 1 PPG acquisition site; 2 (10%) [18,56] studies used forehead-based reflectance pulse-oximetry systems, 1 (5%) [48] used a sternum-based reflectance PPG system, 1 (5%) [53] used a probe at other anatomical sites, and 1 (5%) [16] used an in vitro tissue-vessel phantom rather than an in vivo wearable configuration. Several secondary design features were identified across these primary categories. Three studies (14%) [15,41,47] used force-adjustable or force-regulated platforms, 3 (14%) [43,47,55] used multichannel or multisite PPG acquisition, and 3 (14%) [20,51,55] used multiwavelength reflectance PPG configurations. These secondary categories were nonmutually exclusive because individual systems could incorporate more than 1 design feature. Detailed study-level configurations, fixation strategies, optical settings, and reference measurements are presented in Table 2.

#### Characteristics of Contact-Force and Contact-Pressure Reporting

The representation of contact conditions differed substantially across the included studies. Based on the principal mechanical quantity or descriptor reported in each study, the 21 studies were classified into 4 mutually exclusive categories. As many as 5 (24%) studies [15,41,44,45,54] used force-based representations. Of these, all 5 [15,41,44,45,54] explicitly reported contact force in Newtons. Among them, Teng and Zhang [54] examined discrete applied contact-force levels through theoretical modeling and validation involving human participants. Ten studies (48%) [16,18,20,21,42,49,51,53,55,56] used pressure-based representations reported in physical pressure units, including mm Hg (millimeters of mercury), kPa (kilopascal), or torr. These representations included externally applied or cuff-derived pressure values [20,42,51], preset wristband or strap-pressure levels [21,49], and locally applied probe-skin, sensor-skin, or device-housing pressure [16,18,53,55,56]. Four studies (19%) [43,47,50,52] used nonstandardized sensor-output, normalized, indirect, or categorical representations. These included contact-force–related piezoelectric sensor output without a reported physical unit [43], sequential load-cell readings without conversion to a standardized force or pressure value [47], normalized force divided into predefined ranges [50], and pressure-related waveform-morphology labels [52]. Two studies (10%) [46,48] described sensor-skin contact qualitatively or contextually without directly quantifying the applied force or pressure. One study [49] explicitly reported the sensor-skin contact area. Four studies [20,43,44,47] provided partial sensor, probe, printed circuit board, or device-geometry information but did not report the effective skin-contact area. For the remaining studies, contact-area or probe-geometry information was insufficient or unavailable. Sensor or device dimensions were not assumed to represent effective skin-contact area unless this relationship was explicitly established. Consequently, reported force values could not always be converted into contact pressure or directly compared with pressure values across devices. Although contact force and contact pressure are mechanically related, they were not treated as interchangeable when sufficient contact-area information was unavailable.

#### Characteristics of Multilevel PPG Responses Under Different Contact Force/Pressure

**Figure 2:**
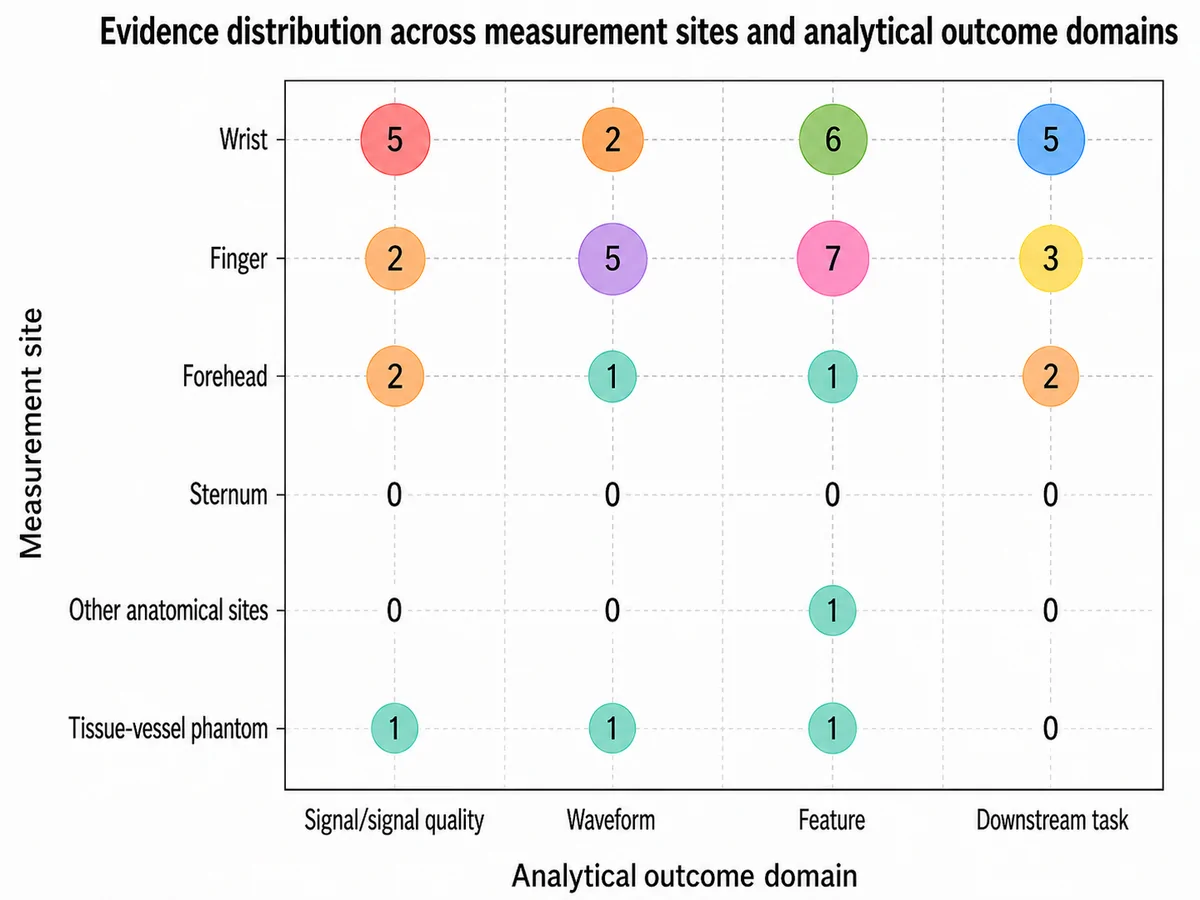
Evidence distribution across measurement sites and analytical outcome domains. Bubble size represents the number of unique studies contributing direct empirical response evidence to each site-outcome combination. Studies could contribute to more than 1 cell. Empty cells indicate that no direct empirical evidence of a response to the contact condition was mapped to that combination.

**Figure 3:**
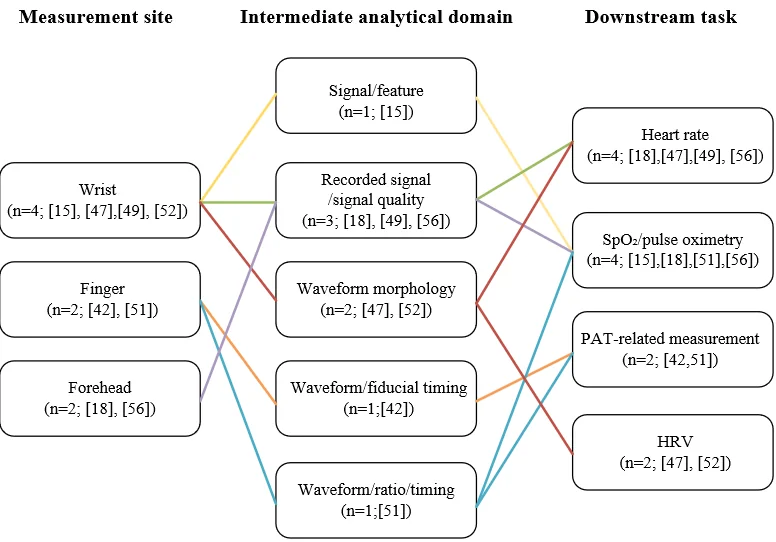
Complete within-study empirical linkages from measurement sites through intermediate photoplethysmography domains to downstream tasks. Each colored pathway represents a single study; branches indicate that a study contributed evidence linking the same pathway to multiple downstream tasks. HRV: heart rate variability; PAT: pulse arrival time; SpO_2_: peripheral oxygen saturation.

**Figure 4:**
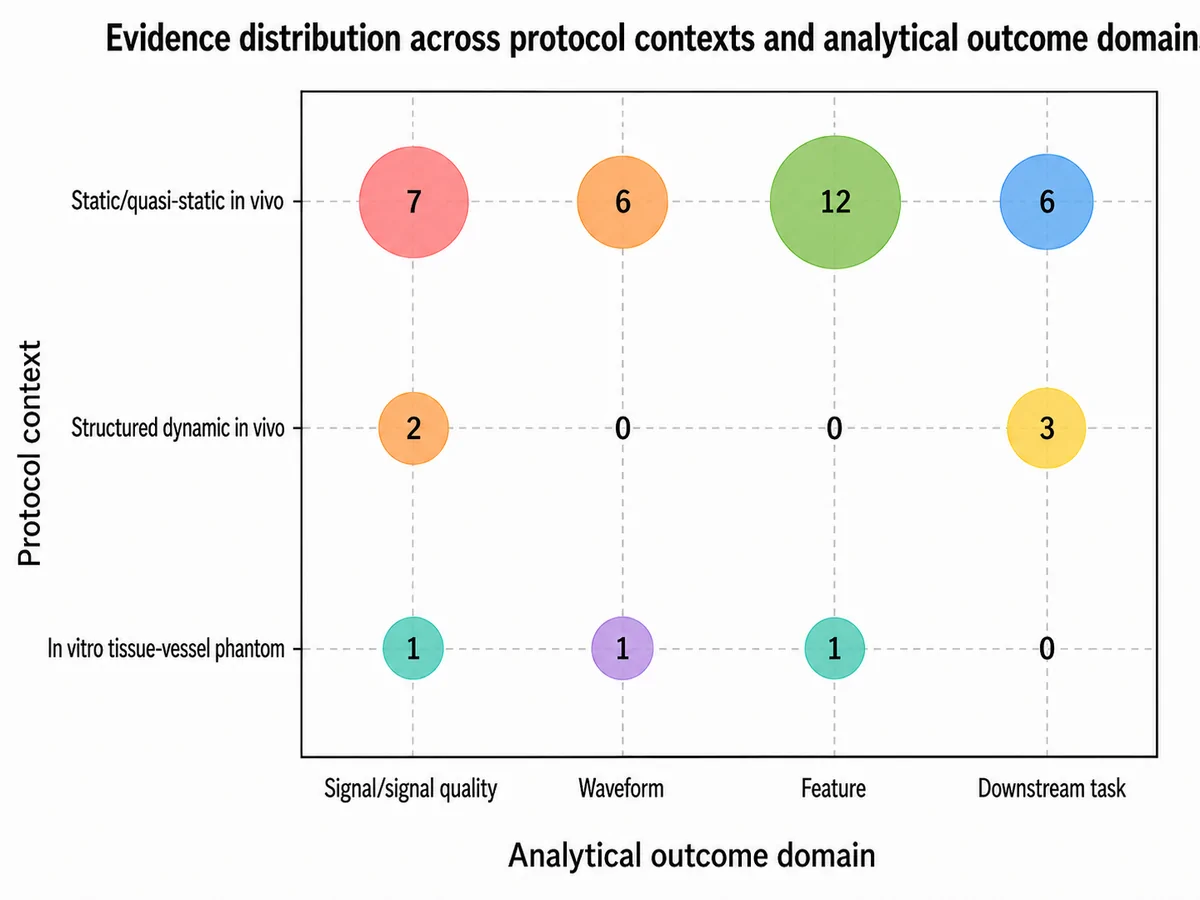
Distribution of direct empirical evidence across principal protocol contexts and analytical outcome domains. Bubble area represents the number of unique studies contributing evidence to each protocol-outcome combination. Each study was assigned to 1 principal protocol category but could contribute to multiple outcome domains. Empty cells indicate that no direct empirical evidence was mapped to that combination.

The reported outcomes spanned signal or signal-quality, waveform, feature, and downstream-task domains. Sixteen studies [15,16,18,20,41-45,47,49-51,53,55,56] contributed direct signal-, waveform-, or feature-level evidence, whereas 10 studies [15,18,21,42,47,49,51,52,54,56] evaluated at least 1 downstream task under different contact conditions. These categories were nonmutually exclusive. Detailed study-level findings are presented below and synthesized across measurement sites, protocol contexts, and analytical domains in Figures 2-4 and Table 4. The studies considered in the empirical linkages in Figure 3 are [15,18,42,47,49,51,52,56].

### Results of Individual Sources of Evidence

#### Overview

To avoid duplicating the detailed study-level tables, the following narrative highlights key findings from individual sources.

#### Contact-Force Control and Sensing Methods

As outlined in Table 1, the included studies can be broadly categorized into 4 methodological groups according to their approaches to contact force and contact pressure in wearable reflectance PPG: active closed-loop force control, direct contact force/pressure measurement, multipoint contact-force monitoring, and indirect contact-force estimation based on PPG signals. These approaches reflect 4 distinct methodological priorities, namely, stabilizing contact conditions, quantifying contact state, characterizing spatial variations in contact, and reducing reliance on additional hardware. Sim et al [41] represented the first direction by developing an active closed-loop force-control platform, through which the analysis could be performed under constant contact conditions, thereby emphasizing signal stability and experimental repeatability. By contrast, Liu et al [43] extended the problem from single-point force regulation to multipoint synchronous sensing, enabling the investigation of relationships between spatial variations in contact state and PPG waveform behavior. Přibil et al [44] further highlighted the direct measurement and quantitative characterization of probe-skin contact force by integrating force-sensitive sensing into the optical probe, which improved the physical interpretability of experimental conditions and facilitated comparison across studies. Fortin et al [45], in turn, explored a different direction by attempting to estimate contact force indirectly from raw PPG signals, thereby reducing dependence on dedicated force-sensing hardware.

#### Measurement Sites and Wearable Configurations

As shown in Table 2, from the perspective of measurement site and device configuration, current reflectance PPG research has moved beyond the simple question of where a sensor should be placed. Instead, it increasingly treats measurement site, wearing form, reference channel, and target task as a coupled system-level design problem. Charlton et al [46] reported that wrist PPG signal quality varied across body postures and sensor heights relative to the heart. Ho et al [47] further showed that this systemic issue also involves contact-force control and reference-channel configuration; by combining a force-adjustable wrist module with a high-perfusion fingertip reference, they more clearly revealed the coupling among measurement site, wearing structure, and reference design. The study by Dresher and Mendelson [18] likewise indicated that the effectiveness of the forehead as a measurement site cannot be understood independently of the specific wearing configuration. Using a forehead reflectance pulse oximetry device consisting of an elastic headband and a custom housing, they systematically evaluated the performance of PPG signals measured from the supraorbital forehead region under different contact pressures and showed that forehead signal quality and measurement reliability during motion are shaped not only by the measurement site itself but also by the combined effects of fixation structure and contact-pressure conditions. Chan et al [48] extended the measurement site from the periphery to the mid-sternum, further indicating that although more central reflectance PPG holds promise for continuous oxygen saturation monitoring, its performance remains constrained by multiple factors, including local perfusion, optical path design, and wearing strategy.

#### Static and Dynamic Study Protocols

As summarized in Table 3, existing protocols can be broadly categorized into static/quasi-static designs and dynamic in vivo designs. Static and quasi-static protocols primarily examined contact-condition–related changes in waveform morphology, signal-to-noise ratio (SNR), and derived features while minimizing motion-related variation. Dynamic protocols examined whether similar response patterns were observed during stepping, walking, running, or other wearable-use conditions. In the dynamic category, Scardulla et al [49] investigated wrist PPG during step exercise at 3 preset contact pressures and showed that contact pressure had a greater impact on heart-rate measurement quality than exercise intensity itself. Scardulla et al [21] later extended this line of work using a more standardized treadmill protocol across 20, 60, and 75 mm Hg and found that 60 mm Hg offered the best overall balance of accuracy and precision, although the optimal pressure still varied across individuals. By contrast, quasi-static and static studies have been more focused on mechanism. Sirkiä et al [20], using a controlled pressure ramp on the fingertip with multiwavelength PPG, showed that externally applied pressure changes not only oscillation amplitudes but also pressure-dependent occlusion behavior and interchannel timing differences, indicating depth-dependent vascular responses. May et al [16], in an in vitro tissue-vessel phantom study with continuously increasing pressure under multiple simulated blood pressure states, demonstrated the existence of an optimal contact-pressure range and further showed that temporal features are relatively robust, whereas amplitude- and geometry-related features are substantially more pressure-sensitive. Ho et al [47] further contributed a quasi-static wrist-finger dual-channel dataset collected under sedentary conditions, in which wrist pressure was gradually adjusted while a fingertip channel was maintained as a high-quality reference; their results showed systematic pressure-dependent transitions in wrist PPG morphology and corresponding degradation in downstream HR and HRV estimation under suboptimal pressure.

#### Contact-Condition–Related Findings in Reflectance PPG

##### Signal and Signal-Quality Responses

As outlined in Table S1 in Multimedia Appendix 5, the included signal-quality studies reported contact-condition–related differences across several PPG dimensions, including signal amplitude, perfusion-related measures, noise, baseline stability, motion artifacts, and morphology-based quality indices. Lambert Cause et al [50] evaluated normalized fingertip force across 5 bins using skewness, kurtosis, relative power, entropy, zero-crossing rate, and perfusion index and found that different signal-quality indices favored different force ranges. May et al [16] demonstrated in vitro that the SNR first increased and then decreased as contact pressure rose, with a clear optimum before full occlusion. He et al [15] quantified the force dependence of high-frequency noise, baseline drift, and motion artifacts, showing that higher force could reduce some motion-related degradation, although not uniformly across all indices. Charlton et al [46] further emphasized that contact pressure should be interpreted jointly with posture, sensor height, and skin contact state when signal quality is assessed at the wrist.

##### Waveform and Feature Responses

As outlined in Table S2 in Multimedia Appendix 5, the included studies reported contact-condition–related differences not only in signal amplitude but also in waveform morphology and derived feature behavior. May et al [16] showed that PPG amplitude and SNR followed an inverted-U pattern as pressure increased. Ho et al [47] revealed that low-force wrist PPG often appeared single-peaked or noise-dominated, whereas moderate force revealed a more canonical 2-peak morphology with a clearer systolic peak, dicrotic notch, and diastolic peak; at still higher force, the waveform could collapse back toward a distorted single-peak shape. Sirkiä et al [20,51] further demonstrated that contact pressure affects both AC and DC components and that wavelength channels respond differently. Grabovskis et al [53] investigated the effect of probe contact pressure on PPG-based conduit artery stiffness assessment and showed that variable probe pressure affected the AC PPG second-derivative peak ratio b/a, a morphology-derived parameter associated with arterial stiffness. Fine and Kaminsky [55] showed that, in reflection pulse oximetry, gamma, a dual-wavelength PPG-derived ratio parameter used in arterial oxygen saturation calculation, increased with increasing applied pressure.

##### Findings on Artifact Susceptibility and Temporal Stability

As outlined in Table S3 in Multimedia Appendix 5, several included studies examined contact conditions in relation to artifact susceptibility and the temporal stability of PPG-derived measurements. At the wavelength level, Sirkiä et al [20,51] found that shorter wavelengths were more pressure-sensitive than red and infrared channels, with earlier onset of occlusion-like behavior and waveform distortion as pressure increased. At the feature level, Sim et al [41] showed that amplitude-dominated indices were more force-sensitive than interval-dominated indices. At the task level, Scardulla et al [49] found that excessively low pressure promoted poor contact and motion artifacts during exercise, whereas excessively high pressure could reduce precision by compressing local microcirculation.

##### Findings for SpO2 and Other Downstream Estimation Tasks

As outlined in Table S4 in Multimedia Appendix 5, a smaller body of evidence examined contact force and contact pressure in relation to downstream physiological estimation tasks beyond signal- and waveform-level outcomes. For SpO_2_ estimation, He et al [15], in a study of wrist reflectance PPG-based oxygen saturation prediction, showed that explicitly incorporating force-related features into an SpO_2_ prediction model improved estimation accuracy, reducing the mean absolute error to 0.8811%. For BP estimation, Chandrasekhar et al [42], in a study using PPG-derived PAT for potential cuffless BP measurement, found that contact-pressure–induced changes in waveform morphology and fiducial-point location shifted PAT_foot_ and PAT_peak_ by 22 (SD 2) ms and 40 (SD 7) ms, respectively. Here, PAT_foot_ refers to PAT estimated from the foot of the PPG waveform, whereas PAT_peak_ refers to PAT estimated from the systolic peak. These timing shifts corresponded to approximately 11 and 20 mm Hg of BP estimation error, respectively, indicating that pressure-induced waveform distortion can directly propagate into BP estimation. For heart-rate estimation, Dresher and Mendelson [56] designed a reflectance pulse oximeter housing with contact-pressure measurement and showed that excessive sensor-skin pressure can impair local blood flow, attenuate or even eliminate PPG signals, and thereby affect pulse-oximetry measurement accuracy. Their dual-housing design improved HR accuracy under large contact-pressure variations. Their findings linked contact-pressure variation with differences in pulse-oximetry and heart-rate performance. Scardulla et al [21,49], in studies evaluating PPG signal performance under different contact-pressure conditions, showed that heart-rate estimation is strongly pressure-dependent, with intermediate pressures generally providing the best group-level performance, whereas both loose and excessively tight configurations degrade accuracy through different mechanisms. Hu et al [52], in wrist-PPG–based HRV estimation under sedentary conditions, demonstrated that even subtle waveform morphology distortions can degrade HRV estimation performance. They showed that morphology-aware modeling reduced the gap between wrist PPG and reference measurements, indicating that pressure-related morphology changes may impair HRV estimation by disturbing beat-to-beat timing and waveform-feature extraction.

#### Nonlinear Force Dependence and Moderate-Force Behavior

Several included studies reported nonlinear or task-dependent response patterns under their respective experimental conditions. As outlined in Table S5 in Multimedia Appendix 5, for example, May et al [16] found that both waveform amplitude and SNR first increased and then decreased as pressure rose. Lambert Cause et al [50] reported that the perfusion index, which is strongly amplitude-dependent, was also highest in lower-to-moderate force ranges before declining at higher force. Their signal-quality indices likewise showed that both low and high extremes were suboptimal, whereas moderate force better preserved waveform detail. Sirkiä et al [20,51] further showed that SpO_2_ estimates remained relatively stable at low pressure but began to rise as pressure approached diastolic and mean arterial pressure, indicating that pressure-induced changes in AC/DC relationships and feature points can bias downstream metrics. Teng and Zhang [54] investigated the effect of sensor contact force on PPG-derived PTT using theoretical modeling and validation involving human participants. By considering the nonlinear pressure-volume relationship of the finger arterial wall, they showed that PTT increased with contact force under positive transmural pressure, reached a maximum near zero transmural pressure, and then remained nearly constant under negative transmural pressure. This finding indicates that sensor contact force can nonlinearly alter PPG-derived timing parameters through changes in local transmural pressure and arterial wall mechanics. Across these studies, nonlinear or inverted-U-like response patterns were reported under the respective experimental conditions. Intermediate contact levels were frequently associated with better signal quality, waveform clarity, or task performance than the lowest or highest tested levels. Importantly, the location and shape of this optimum are not fixed but vary with the physiological target, wavelength, measurement site, and feature type.

#### Synthesis of Results

##### Evidence Distribution Across Measurement Sites and Analytical Outcome Domains

Figure 2 maps direct empirical response evidence across measurement sites and analytical outcome domains. At the wrist, direct empirical evidence included 5 signal- or signal-quality-level studies [15,21,41,43,49], 2 waveform-level studies [43,47], 6 feature-level studies [15,43,45,47,52,55], and 5 downstream-task studies evaluating SpO_2_, HR, or HRV [15,21,47,49,52]. At the finger, 2 studies reported signal-level responses [44,50], 5 examined AC/DC behavior, pulse shape, or waveform fiducial points [20,42,44,51,55], 7 evaluated timing-, morphology-, perfusion-, or ratio-based features [20,42,44,45,50,51,55], and 3 assessed SpO_2_, PAT, or PTT as downstream tasks [42,51,54]. At the forehead, 2 studies reported signal- or signal-quality-level responses [18,56], 1 examined waveform-level responses [56], 1 evaluated feature-level responses [55], and 2 assessed HR or pulse-oximetry performance [18,56]. Evidence from other sites was limited: 1 study [53] at other anatomical sites reported morphology- and derivative-based feature responses, whereas the tissue-vessel phantom study [16] contributed signal-, waveform-, and feature-level evidence across progressively increased pressure conditions. No direct empirical contact-condition response was mapped for the sternum study [48]. As individual studies could contribute to more than 1 measurement-site or analytical-domain category, the categories were nonmutually exclusive. The figure summarizes the distribution of evidence but does not determine whether intermediate PPG responses and downstream tasks were connected within the same study; these complete within-study linkages are presented in Figure 3.

##### Evidence Linkages Across Measurement Sites, Intermediate Analytical Domains, and Downstream Tasks

At the wrist, He et al [15] linked contact-force–responsive signal-quality indices, including high-frequency noise, baseline drift, and motion artifact, together with contact-force information, to PPG-based SpO_2_ prediction. Ho et al [47] linked pressure-related changes in waveform morphology, including the systolic peak, dicrotic notch, and diastolic peak, to HR and HRV estimation. Scardulla et al [49] linked contact stability and motion-artifact susceptibility during exercise to PPG-derived HR evaluation. Hu et al [52] used raw wrist PPG waveforms and pressure-related morphology labels to estimate NN intervals and beat count for HRV computation. At the finger, Chandrasekhar et al [42] linked changes in waveform-foot and systolic-peak timing to PAT_foot_ and PAT_peak_. Sirkiä et al [51] linked AC/DC components, wavelength-dependent waveform responses, amplitude- and perfusion-related characteristics, and pulse-foot and pulse-peak timing to SpO_2_, PAT_foot_, and PAT_peak_. At the forehead, Dresher and Mendelson [18] linked contact-pressure–related PPG signal quality and measurement reliability during walking to HR and SpO_2_ performance. In a related housing study, Dresher and Mendelson [56] linked pressure-related differences in forehead PPG amplitude and signal quality to SpO_2_ and HR measurement performance. Overall, the complete within-study linkages involved signal-quality indices and contact-force information for SpO_2_ prediction [15], waveform morphology for HR and HRV estimation [47,52], dynamic signal stability for HR evaluation [49], fiducial-point timing for PAT measurement [42], AC/DC and wavelength-dependent responses for SpO_2_ and PAT calculation [51], and forehead PPG signal quality and amplitude for HR and pulse-oximetry performance [18,56].

##### Evidence Distribution Across Protocol Contexts and Outcome Domains

Under static or quasi-static in vivo conditions, direct empirical evidence included 7 studies [15,41,43-45,50,56] reporting signal- or signal-quality-level responses, 6 [20,42,44,47,51,55] examining waveform-level changes, 12 [15,20,42-45,47,50-53,55] evaluating feature-level responses, and 7 [15,42,47,51,52,54,56] assessing downstream tasks. Under structured dynamic conditions, 2 studies [18,49] reported signal- or signal-quality-level responses, whereas 3 [18,21,49] evaluated downstream tasks, primarily HR-related performance. In the in vitro tissue-vessel phantom protocol, 1 study [16] provided signal-, waveform-, and feature-level evidence across controlled pressure conditions. Overall, Figure 4 maps directly measured or calculated PPG responses across principal protocol contexts and analytical outcome domains. The mapped distribution indicates that waveform- and feature-level evidence was concentrated in static or quasi-static in vivo protocols, whereas structured dynamic protocols mainly addressed signal behavior and downstream HR performance.

#### Study-Level Coverage Across Methodological and Analytical Domains

Table 4 provides a study-level overview of the methodological and analytical domains covered by the 21 included studies. Measurement site and principal protocol context were identifiable for all studies. Participant characteristics and contact-condition representation were more consistently documented than effective contact area or probe geometry. Sensor, printed circuit board, load-cell, or device dimensions without sufficient information on the effective skin-contact interface were classified as partial rather than sufficient reporting. Analytical coverage varied across studies, with some studies addressing multiple signal-, waveform-, feature-, and downstream-task domains. Overall, the matrix highlights variation in methodological reporting and analytical breadth rather than differences in study quality.

**Table 4:** Overview of the methodological and analytical domains covered by the 21 included studies.

Study	Study context			Methodological reporting				Analytical coverage			
	Site	Protocol	Participants		Contact-condition representation	Geometry/contact area	Signal/signal quality		Waveform	Feature	Downstream task
He et al [15]	Wrist	Static/quasi-static in vivo	Sufficient		Sufficient	Insufficient or unavailable	Assessed		Not assessed	Assessed	Assessed
May et al [16]	Tissue-vessel phantom	In vitro tissue-vessel phantom	N/A^a^		Sufficient	Insufficient or unavailable	Assessed		Assessed	Assessed	Not assessed
Sim et al [41]	Wrist	Static/quasi-static in vivo	Sufficient		Sufficient	Insufficient or unavailable	Assessed		Assessed	Not assessed	Not assessed
Chandrasekhar et al [42]	Finger	Static/quasi-static in vivo	Sufficient		Sufficient	Insufficient or unavailable	Not assessed		Assessed	Assessed	Assessed
Dresher et al [18]	Forehead	Structured dynamic in vivo	Insufficient or unavailable		Sufficient	Insufficient or unavailable	Assessed		Not assessed	Not assessed	Assessed
Sirkiä et al [20]	Finger	Static/quasi-static in vivo	Sufficient		Sufficient	Partial or indirect	Not assessed		Assessed	Assessed	Not assessed
Scardulla et al [21]	Wrist	Structured dynamic in vivo	Sufficient		Sufficient	Insufficient or unavailable	Assessed		Not assessed	Not assessed	Assessed
Liu et al [43]	Wrist	Static/quasi-static in vivo	Insufficient or unavailable		Partial or indirect	Partial or indirect	Assessed		Assessed	Assessed	Not assessed
Přibil et al [44]	Finger	Static/quasi-static in vivo	Sufficient		Sufficient	Partial or indirect	Assessed		Not assessed	Assessed	Not assessed
Fortin et al [45]	Multiple sites	Static/quasi-static in vivo	Sufficient		Sufficient	Insufficient or unavailable	Assessed		Not assessed	Assessed	Not assessed
Charlton et al [46]	Wrist	Observational	Sufficient		Insufficient or unavailable	Insufficient or unavailable	Assessed		Not assessed	Not assessed	Not assessed
Ho et al [47]	Multiple sites	Static/quasi-static in vivo	Sufficient		Partial or indirect	Partial or indirect	Not assessed		Assessed	Assessed	Assessed
Chan et al [48]	Sternum	Observational	Insufficient or unavailable		Insufficient or unavailable	Insufficient or unavailable	Not assessed		Not assessed	Not assessed	Assessed
Scardulla et al [49]	Wrist	Structured dynamic in vivo	Sufficient		Sufficient	Sufficient	Assessed		Not assessed	Not assessed	Assessed
Lambert Cause et al [50]	Finger	Static/quasi-static in vivo	Insufficient or unavailable		Partial or indirect	Insufficient or unavailable	Assessed		Not assessed	Assessed	Not assessed
Sirkiä et al [51]	Finger	Static/quasi-static in vivo	Sufficient		Sufficient	Insufficient or unavailable	Assessed		Assessed	Assessed	Assessed
Hu et al [52]	Wrist	Static/quasi-static in vivo	Sufficient		Partial or indirect	Insufficient or unavailable	Not assessed		Assessed	Assessed	Assessed
Grabovskis et al [53]	Other anatomical site	Static/quasi-static in vivo	Sufficient		Sufficient	Insufficient or unavailable	Not assessed		Assessed	Assessed	Assessed
Teng and Zhang [54]	Finger	Static/quasi-static protocol with theoretical modeling and validation of human participants	Sufficient		Sufficient	Insufficient or unavailable	Not assessed		Not assessed	Not assessed	Assessed
Fine and Kaminsky [55]	Multiple sites	Static/quasi-static in vivo	Insufficient or unavailable		Sufficient	Insufficient or unavailable	Assessed		Assessed	Assessed	Assessed
Dresher and Mendelson [56]	Forehead	Static/quasi-static in vivo	Sufficient		Sufficient	Insufficient or unavailable	Assessed		Assessed	Not assessed	Assessed

^a^N/A: not applicable.

## Discussion

### Principal Findings

This scoping review identified 21 studies that examined how contact force or contact pressure was controlled, measured, estimated, or characterized in wearable or wearable-relevant reflectance PPG, together with the reported signal-, waveform-, feature-, and task-level outcomes under different contact conditions [15,16,18,20,21,41-56]. Previous reviews have synthesized the physiological basis of PPG, waveform analysis, signal-processing methods, and downstream applications such as HR, BP, oxygen saturation, and arrhythmia detection [7-10,22-29]; wearable-device architectures and configurations [30-34]; and signal-quality challenges, datasets, and analytical tools [7,29,35-37]. Within these broader reviews, contact pressure, wearing tightness, and sensor-skin interface stability have generally been discussed as acquisition or signal-quality considerations rather than as the central framework for organizing the evidence [29-32]. Building on this literature, our review places contact force and contact pressure at the center of the synthesis, distinguishes these 2 mechanical quantities, and integrates evidence on their measurement, control, representation, and reporting with outcomes across multiple levels of PPG analysis. It also maps the distribution of evidence across anatomical sites, device configurations, populations, experimental settings, and protocol durations.

The mapped evidence indicates that contact force and contact pressure are important measurement conditions associated with variability in wearable reflectance PPG observations and should not be treated solely as minor wearing-related factors. Across the included studies, different contact conditions were reported in relation to differences in signal amplitude, signal quality, motion susceptibility, waveform morphology, feature stability, and downstream physiological estimation [15,16,18,20,21,41,42,46-56]. However, these responses were not uniform across feature categories or experimental settings. Amplitude-, morphology-, and derivative-based parameters generally varied more across contact conditions than some timing-related features, suggesting that feature stability may depend on the type of parameter examined [16,41,53]. The reviewed studies further indicated that contact-condition–related responses varied across measurement sites, wavelengths, fixation strategies, postures, and motion conditions, rather than following a single relationship that could be generalized across devices or settings [18,20,21,46-48,51]. Their practical relevance also appeared to differ by task: applications that rely on waveform morphology, amplitude relationships, or precise fiducial-point localization may require more task-specific evaluation than applications based primarily on preserved periodicity [15,20,21,42,49,51,52]. Compared with previous broad reviews of wearable PPG, this review therefore provides a focused methodological and conceptual map of the sensor-skin mechanical interface. It also identifies incomplete mechanical reporting, limited real-world validation, restricted population and measurement-site diversity, and incomplete linkage between intermediate signal characteristics and downstream estimation as important priorities for future research.

### Limitations

#### Inconsistent Reporting of Contact Force and Contact Pressure

Despite growing interest, the mapped evidence remains methodologically heterogeneous [15,16,18,20,21,41-56]. A major limitation is the lack of a standardized framework for representing, measuring, and reporting contact force and contact pressure [15,16,18,20,21,41-56]. Importantly, contact force and contact pressure are mechanically related but not interchangeable: contact force describes the total load applied to the sensor-skin interface, whereas contact pressure depends on how that load is distributed over the effective contact area [15,16,20,21,41-47,50-56]. Across studies, contact conditions were described using different units or concepts, including Newtons, mm Hg, normalized force units, cuff pressure, preset strap levels, inferred tightness, or qualitative descriptions of skin contact [15,16,18,20,21,41-56]. This heterogeneity limits direct comparison and the development of quantitative design guidance [15,16,20,21,41-47,49-56]. It also limits the transferability of terms such as “moderate force” or “optimal pressure” across wearable configurations [16,20,21,47,49-51,54]. In particular, when studies report only force without specifying the effective contact area, probe geometry, or active sensing area, the corresponding contact pressure cannot be reliably derived [15,41,43-45,47,50,52]. Even when similar terms are used, the underlying physical meaning may differ because reported pressure can depend on probe geometry, active sensing area, tissue compliance, or the way force is converted into pressure [16,20,21,47,49-51,53,54]. A more standardized reporting convention would therefore be an important step toward improving comparability and reproducibility [57,58]. Future studies should report the effective sensor-skin contact area or probe geometry when reporting contact force, or directly report contact pressure in standardized units such as mm Hg or kPa [57,58]. This would improve reproducibility, cross-study comparability, and practical translation for wearable PPG device design.

#### Limited Real-World and Longitudinal Validation

A major evidence gap is the limited validation of contact conditions during prolonged and unsupervised wearable use. Most included studies used small samples, short recordings, highly structured protocols, static or quasi-static tasks, or predefined pressure conditions [16,18,20,21,41,42,44,45,47,49-51,53-56]. These designs are informative for characterizing responses under controlled conditions, but they provide limited evidence about the variability encountered during everyday wear. During free-living use, the sensor-skin interface may vary with posture transitions, arm movement, sweating, changes in skin hydration, tissue deformation, strap displacement, repeated device removal and reattachment, and prolonged wear [14,18,21,46,48,49]. Accordingly, the available laboratory evidence may not capture the full range or temporal instability of contact conditions in natural settings [14,18,21,46,49]. Current evidence does not establish whether a contact condition measured at the start of a recording remains stable over several hours, whether users reproduce similar conditions after repeated donning, or whether contact-related variability is consistent across days. Short supervised walking, stepping, or treadmill protocols should therefore be distinguished from longitudinal or free-living validation. Future studies should jointly document contact force or pressure, motion, posture, wear duration, and contextual factors rather than evaluating contact conditions only under isolated, idealized protocols [14,21,46,47,49,58]. Recent benchmarking efforts, including the QUMPHY D4 report [58], also indicate the value of standardized benchmark datasets in addition to algorithm development. For wearable reflectance PPG, future public datasets should document acquisition context more systematically, including site, device configuration, fixation strategy, wear duration, and other factors that may be associated with signal quality and estimation performance [46,47,58]. Repeated-measures studies should report within-session drift, between-day reproducibility, reattachment procedures, strap or fastening settings, and the frequency of contact-related signal-quality failures.

#### Gap Between Signal-Level Changes and Task-Level Estimation Bias

As shown in Figure 3, complete within-study pathways connecting measurement site, an intermediate signal-, waveform-, or feature-level domain, and a downstream task were identified in 8 studies [15,18,42,47,49,51,52,56]. The remaining studies did not report a complete site-intermediate-domain-downstream-task pathway [16,20,21,41,43-46,48,50,53-55]. This distinction is important because signal or feature differences observed under varying contact conditions do not necessarily correspond to clinically or algorithmically meaningful estimation differences. Their practical relevance is likely to depend on the target task, the signal properties used by the algorithm, and the selected reference method. Conversely, task-level differences should not be attributed solely to contact conditions without also considering motion, wavelength, device configuration, reference-measurement uncertainty, and participant characteristics. Future studies should therefore evaluate contact conditions, intermediate signal or feature behavior, and task-level estimation error within the same experimental protocol and against an appropriate reference standard. Stratified analyses across contact conditions, together with sensitivity or feature-ablation analyses, may help identify which signal properties are most closely associated with task-specific performance differences [15,21,42,49,52,57,58].

#### Limited Population and Measurement-Site Diversity

A further evidence gap concerns population and measurement-site diversity. The evidence was concentrated at the wrist and finger: 10 [15,21,41,43,45-47,49,52,55] of the 21 included studies involved wrist-based configurations, and 9 [20,42,44,45,47,50,51,54,55] involved finger measurements, whereas only 3 [18,55,56] examined the forehead, 1 [48] examined the sternum, and 1 [53] examined another anatomical site; these categories were nonmutually exclusive. Given differences in anatomical structure, device geometry, fixation strategy, and optical measurement configuration across sites, numerical pressure ranges reported at the wrist or finger should not be assumed to apply directly to the forehead, sternum, ear, or other anatomical locations [18,46-48,55,59,60]. Most human studies recruited healthy volunteers [18,20,21,41,42,44,45,47,49-54,56], leaving limited direct evidence for older adults, children, patients with cardiovascular or peripheral vascular conditions, and individuals with edema or impaired perfusion. Previous studies and systematic reviews have reported differences in pulse-oximetry accuracy across skin-pigmentation groups, particularly under lower oxygen-saturation conditions [61-64]. However, the included contact-condition literature did not adequately characterize whether skin pigmentation modifies the relationships between contact conditions and wearable reflectance PPG outcomes. More broadly, the reported specificity of reflectance PPG and SpO_2_ estimation to participant and measurement-site characteristics suggests that population and site characteristics should be considered when interpreting contact-condition–related findings [59,60]. Future studies should report participant age, sex distribution, health status, the method used to characterize skin tone or pigmentation, and relevant vascular or tissue characteristics more consistently. Harmonized multisite protocols are needed to determine whether reported contact-condition responses are site-specific or reproducible across anatomical locations. Findings derived mainly from healthy adults and common peripheral sites should therefore be generalized cautiously to broader populations and other anatomical locations [46,59-64].

#### Scope Boundaries for Transmission-Mode and Hybrid PPG Systems

The focus on wearable or wearable-relevant reflectance PPG represents a deliberate scope boundary of this review. Accordingly, the mapped findings should be applied cautiously to transmission-mode and hybrid reflection-transmission PPG systems. Transmission-mode PPG remains important in wearable pulse oximetry, particularly in finger-clip, ring-type, earlobe, and in-ear devices, in which the light source and photodetector are positioned on opposite sides of relatively thin tissue [30,65-67]. These configurations may provide strong pulsatile signals for SpO_2_ monitoring, but they involve different anatomical constraints and fixation mechanisms, including clips, rings, and in-ear fittings. Differences in optical path, contact area, tissue compression, and fixation mean that reported contact-condition responses may not be directly comparable across optical modes. Numerical force or pressure ranges and response patterns mapped in reflectance studies should therefore not be transferred directly to transmission-mode or hybrid devices. Several methodological principles remain relevant across optical modes, including distinguishing force from pressure and documenting geometry, effective contact area, fixation, and calibration. For hybrid systems, the reflectance and transmission interfaces should be described separately rather than summarized using a single wearing-tightness descriptor. For each sensing interface, studies should report the measurement site, optical mode, wavelength, emitter-detector geometry, fixation method, effective contact area, and contact force or pressure. Direct comparative studies, and potentially a separate evidence synthesis focused on transmission-mode and hybrid systems, are needed before cross-mode recommendations can be established.

#### Methodological Limitations and Scope of the Review

This review also has methodological limitations related to its scope and review process. Although the review followed a scoping review framework and was reported with reference to PRISMA-ScR and PRISMA-S [38-40], the protocol was not formally registered or publicly available. The search strategy was developed by the review authors and refined iteratively, but it did not undergo formal peer review by an information specialist or librarian. In addition, only English-language peer-reviewed journal articles and conference proceedings with accessible full texts were included, whereas non-English studies, non-peer-reviewed sources, conference abstracts, preprints, technical reports, theses, and other gray literature were excluded. Although 4 major databases were searched, publication bias and incomplete database coverage cannot be fully ruled out. Finally, because this was a scoping review, no formal risk-of-bias assessment, critical appraisal, or quantitative meta-analysis was performed; therefore, the findings should be interpreted as a structured mapping and qualitative synthesis of the available evidence rather than as a quantitative estimate of effect size or certainty of evidence.

### Conclusions

Wearable reflectance PPG has become an important sensing technology for mHealth. As wearable PPG moves from controlled settings to real-world use, measurement quality varies with anatomical site, skin and tissue characteristics, motion, and the sensor-skin interface. This scoping review identified a multilevel relationship between contact conditions at the sensor-skin interface and wearable PPG outcomes. Differences in contact conditions were associated with changes in signal characteristics and derived features and, in a smaller body of studies, with differences in downstream physiological estimates such as HR, oxygen saturation, PTT or PAT, blood pressure–related indicators, and HRV. Therefore, contact force should not be treated as a minor wearing-related factor because contact-condition–related differences may extend from PPG signal acquisition to derived features and, in some studies, to downstream physiological estimation. Current evidence remains limited by short-term controlled study designs, inconsistent reporting of force or pressure conditions, insufficient description of contact area and probe geometry, and limited validation in long-term, unsupervised, or free-living settings. Only a few studies have directly linked contact-condition–related signal or feature differences with the robustness of downstream estimation models. Future research should therefore adopt standardized reporting of contact force or pressure, probe geometry, contact area, body site, wavelength, and fixation strategy. Long-term real-world validation, force-aware signal-quality assessment, robustness testing, adaptive attachment design, and context-aware modeling will be essential for improving the reliability and comparability of wearable PPG systems.

## Data Availability

All data extracted and charted in this scoping review are provided in Multimedia Appendices 4 and 5. No additional datasets were generated.

## Appendix

Multimedia Appendix 1 PRISMA-ScR (Preferred Reporting Items for Systematic Reviews and Meta-Analyses extension for Scoping Reviews) checklist and explanation.

Multimedia Appendix 2 PRISMA-S (Preferred Reporting Items for Systematic Reviews and Meta-Analyses literature search extension) checklist.

Multimedia Appendix 3 Search strategy and associated keywords.

Multimedia Appendix 4 Baseline characteristics of the included studies with extracted contact-force/contact-pressure information.

Multimedia Appendix 5 Supporting evidence tables for contact-condition–related findings in wearable reflectance photoplethysmography.

## Abbreviations

AC: alternating current
BP: blood pressure
DC: direct current
HR: heart rate
HRV: heart rate variability
kPa: kilopascal
mHealth: mobile health
mm Hg: millimeters of mercury
PAT: pulse arrival time
PPG: photoplethysmography
PRISMA-S: Preferred Reporting Items for Systematic Reviews and Meta-Analyses literature search extension
PRISMA-ScR: Preferred Reporting Items for Systematic Reviews and Meta-Analyses extension for Scoping Reviews
PTT: pulse transit time
SNR: signal-to-noise ratio
SpO2: peripheral oxygen saturation
