# Supplementary material for: Mechanical Contact Conditions in Wearable Reflectance Photoplethysmography: Scoping Review
Source: JMIR Mhealth Uhealth. 2026 Aug 24;14:e99333. doi: 10.2196/99333 (PMC13550977; doi:10.2196/99333)
Supplement: Supplementary file 3 [file mhealth_v14i1e99333_app3.docx]

**Multimedia Appendix 3. Search strategy and associated keywords**

| **Database** | **Search strategy** | **Search results** |
| --- | --- | --- |
| **PubMed** | (  (  "Photoplethysmography"[Mesh]  OR "Oximetry"[Mesh]  OR photoplethysmograph*[Title/Abstract]  OR photoplethysmogram*[Title/Abstract]  OR photoplethysmographic[Title/Abstract]  OR PPG[Title/Abstract]  OR "pulse oximetr*"[Title/Abstract]  OR "pulse oximeter*"[Title/Abstract]  OR oximeter*[Title/Abstract]  OR "optical pulse"[Title/Abstract]  OR "pulse wave"[Title/Abstract]  )  AND  (  "Wearable Electronic Devices"[Mesh]  OR reflectance[Title/Abstract]  OR "reflectance mode"[Title/Abstract]  OR "reflectance-mode"[Title/Abstract]  OR reflective*[Title/Abstract]  OR wearable*[Title/Abstract]  OR "wearable device*"[Title/Abstract]  OR "wearable sensor*"[Title/Abstract]  OR "wearable monitor*"[Title/Abstract]  OR "wearable system*"[Title/Abstract]  OR "body-worn"[Title/Abstract]  OR "skin-mounted"[Title/Abstract]  OR "skin sensor*"[Title/Abstract]  OR "wrist-worn"[Title/Abstract]  OR wrist[Title/Abstract]  OR wristband*[Title/Abstract]  OR watch[Title/Abstract]  OR smartwatch*[Title/Abstract]  OR "smart watch*"[Title/Abstract]  OR ring[Title/Abstract]  OR "smart ring*"[Title/Abstract]  OR finger*[Title/Abstract]  OR fingertip*[Title/Abstract]  OR thumb*[Title/Abstract]  OR forehead[Title/Abstract]  OR "head-worn"[Title/Abstract]  OR "head-mounted"[Title/Abstract]  OR headband*[Title/Abstract]  OR temple[Title/Abstract]  OR supraorbital[Title/Abstract]  OR ear[Title/Abstract]  OR "ear-worn"[Title/Abstract]  OR "in-ear"[Title/Abstract]  OR earlobe*[Title/Abstract]  OR auricular[Title/Abstract]  OR concha[Title/Abstract]  OR "ear canal"[Title/Abstract]  OR earbud*[Title/Abstract]  OR hearable*[Title/Abstract]  OR earphone*[Title/Abstract]  OR earpiece*[Title/Abstract]  OR sternum[Title/Abstract]  OR sternal[Title/Abstract]  OR chest[Title/Abstract]  OR arm[Title/Abstract]  OR forearm[Title/Abstract]  )  AND  (  "contact force"[Title/Abstract]  OR "contact forces"[Title/Abstract]  OR "contact pressure"[Title/Abstract]  OR "external pressure"[Title/Abstract]  OR "applied pressure"[Title/Abstract]  OR "applied force"[Title/Abstract]  OR "probe pressure"[Title/Abstract]  OR "probe force"[Title/Abstract]  OR "sensor pressure"[Title/Abstract]  OR "sensor force"[Title/Abstract]  OR "skin pressure"[Title/Abstract]  OR "interface pressure"[Title/Abstract]  OR "interface force"[Title/Abstract]  OR "normal force"[Title/Abstract]  OR "normal pressure"[Title/Abstract]  OR "contact load"[Title/Abstract]  OR "loading force"[Title/Abstract]  OR "contact stress"[Title/Abstract]  OR "wearing pressure"[Title/Abstract]  OR "wearing force"[Title/Abstract]  OR "wearing tightness"[Title/Abstract]  OR "strap pressure"[Title/Abstract]  OR "strap force"[Title/Abstract]  OR "strap tightness"[Title/Abstract]  OR "strap tension"[Title/Abstract]  OR "band tightness"[Title/Abstract]  OR "wristband tightness"[Title/Abstract]  OR tightness[Title/Abstract]  OR "device fit"[Title/Abstract]  OR "wearable fit"[Title/Abstract]  OR "skin contact"[Title/Abstract]  OR "sensor contact"[Title/Abstract]  OR "probe contact"[Title/Abstract]  OR "contact condition*"[Title/Abstract]  OR "contact state"[Title/Abstract]  OR "sensor-skin interface"[Title/Abstract]  OR "skin-sensor interface"[Title/Abstract]  OR "probe-skin interface"[Title/Abstract]  OR "sensor-skin contact"[Title/Abstract]  OR "skin-sensor contact"[Title/Abstract]  OR "optical coupling"[Title/Abstract]  OR "mechanical coupling"[Title/Abstract]  OR "coupling pressure"[Title/Abstract]  OR "contact area"[Title/Abstract]  OR compression[Title/Abstract]  OR "skin compression"[Title/Abstract]  OR "tissue compression"[Title/Abstract]  OR "vascular compression"[Title/Abstract]  OR "vascular occlusion"[Title/Abstract]  OR "transmural pressure"[Title/Abstract]  OR "skin indentation"[Title/Abstract]  OR "indentation force"[Title/Abstract]  )  AND  (  "Signal-To-Noise Ratio"[Mesh]  OR "Artifacts"[Mesh]  OR "Heart Rate"[Mesh]  OR "Oxygen Saturation"[Mesh]  OR "Blood Pressure"[Mesh]  OR "Blood Pressure Determination"[Mesh]  OR "Perfusion Index"[Mesh]  OR "Pulse Wave Analysis"[Mesh]  OR "Calibration"[Mesh]  OR "Reproducibility of Results"[Mesh]  OR "signal quality"[Title/Abstract]  OR "signal-to-noise"[Title/Abstract]  OR SNR[Title/Abstract]  OR artifact*[Title/Abstract]  OR "motion artifact*"[Title/Abstract]  OR noise[Title/Abstract]  OR "waveform morpholog*"[Title/Abstract]  OR waveform[Title/Abstract]  OR morphology[Title/Abstract]  OR "pulse waveform"[Title/Abstract]  OR "feature extraction"[Title/Abstract]  OR feature*[Title/Abstract]  OR "fiducial point*"[Title/Abstract]  OR "foot point"[Title/Abstract]  OR "systolic peak"[Title/Abstract]  OR "diastolic peak"[Title/Abstract]  OR "dicrotic notch"[Title/Abstract]  OR "AC/DC"[Title/Abstract]  OR "AC component*"[Title/Abstract]  OR "DC component*"[Title/Abstract]  OR "perfusion index"[Title/Abstract]  OR "pulse amplitude"[Title/Abstract]  OR "pulse area"[Title/Abstract]  OR "rise time"[Title/Abstract]  OR "reflection index"[Title/Abstract]  OR "stiffness index"[Title/Abstract]  OR "augmentation index"[Title/Abstract]  OR "pulse arrival time"[Title/Abstract]  OR PAT[Title/Abstract]  OR "pulse transit time"[Title/Abstract]  OR PTT[Title/Abstract]  OR "heart rate"[Title/Abstract]  OR HR[Title/Abstract]  OR "heart rate variability"[Title/Abstract]  OR HRV[Title/Abstract]  OR SpO2[Title/Abstract]  OR "oxygen saturation"[Title/Abstract]  OR "blood pressure"[Title/Abstract]  OR "blood pressure estimation"[Title/Abstract]  OR "blood pressure monitoring"[Title/Abstract]  OR "physiological estimation"[Title/Abstract]  OR "estimation accuracy"[Title/Abstract]  OR "measurement accuracy"[Title/Abstract]  OR calibrat*[Title/Abstract]  OR error*[Title/Abstract]  )  )  AND English[Language]  AND "Humans"[Mesh] | 165 |
| **IEEE Xplore** | ( ( "All Metadata":"photoplethysmography" OR "All Metadata":"photoplethysmogram" OR "All Metadata":"photoplethysmograph" OR "All Metadata":"PPG" OR "All Metadata":"pulse oximetry" OR "All Metadata":"pulse oximeter" ) AND ( "All Metadata":"reflectance" OR "All Metadata":"reflectance mode"  OR "All Metadata":"reflective" OR "All Metadata":"wearable" OR "All Metadata":"wearable device" OR "All Metadata":"body-worn" OR "All Metadata":"skin-mounted" OR "All Metadata":"wrist"  OR "All Metadata":"smartwatch"  OR "All Metadata":"smart watch"  OR "All Metadata":"ring"  OR "All Metadata":"smart ring"  OR "All Metadata":"finger" OR "All Metadata":"fingertip" OR "All Metadata":"thumb" OR "All Metadata":"forehead" OR "All Metadata":"temple" OR "All Metadata":"supraorbital" OR "All Metadata":"ear" OR "All Metadata":"ear-worn" OR "All Metadata":"in-ear" OR "All Metadata":"earlobe" OR "All Metadata":"concha" OR "All Metadata":"ear canal" OR "All Metadata":"sternum" OR "All Metadata":"sternal" OR "All Metadata":"chest" ) AND ( "All Metadata":"contact force" OR "All Metadata":"contact pressure" OR "All Metadata":"external pressure" OR "All Metadata":"applied pressure" OR "All Metadata":"probe pressure" OR "All Metadata":"sensor pressure" OR "All Metadata":"skin pressure" OR "All Metadata":"interface pressure" OR "All Metadata":"wearing pressure" OR "All Metadata":"strap pressure" OR "All Metadata":"strap tightness" OR "All Metadata":"tightness" OR "All Metadata":"contact condition" OR "All Metadata":"contact state" OR "All Metadata":"skin contact" OR "All Metadata":"sensor-skin interface" OR "All Metadata":"skin-sensor interface" OR "All Metadata":"probe-skin interface" OR "All Metadata":"optical coupling" OR "All Metadata":"mechanical coupling" OR "All Metadata":"contact area" OR "All Metadata":"tissue compression" OR "All Metadata":"vascular compression" OR "All Metadata":"compression" ) AND ( "All Metadata":"signal quality" OR "All Metadata":"signal-to-noise" OR "All Metadata":"SNR" OR "All Metadata":"artifact" OR "All Metadata":"motion artifact" OR "All Metadata":"waveform morphology" OR "All Metadata":"morphology" OR "All Metadata":"feature extraction" OR "All Metadata":"fiducial point" OR "All Metadata":"AC component" OR "All Metadata":"DC component" OR "All Metadata":"perfusion index" OR "All Metadata":"heart rate" OR "All Metadata":"heart rate variability" OR "All Metadata":"HRV" OR "All Metadata":"oxygen saturation" OR "All Metadata":"SpO2" OR "All Metadata":"blood pressure" OR "All Metadata":"pulse arrival time" OR "All Metadata":"pulse transit time" OR "All Metadata":"estimation accuracy" OR "All Metadata":"calibration" ) ) | 181 |
| **Scopus** | (  TITLE-ABS-KEY(  photoplethysmograph* OR photoplethysmogram* OR photoplethysmographic OR PPG  OR "pulse oximetr*" OR "pulse oximeter*" OR oximeter* OR "optical pulse" OR "pulse wave"  )  AND TITLE-ABS-KEY(  reflectance OR "reflectance mode" OR "reflectance-mode" OR reflective* OR wearable*  OR "wearable device*" OR "wearable sensor*" OR "wearable monitor*" OR "wearable system*"  OR "body-worn" OR "skin-mounted" OR "skin sensor*" OR "wrist-worn" OR wrist OR wristband*  OR watch OR smartwatch* OR "smart watch*" OR ring OR "smart ring*" OR finger* OR fingertip*  OR thumb* OR forehead OR "head-worn" OR "head-mounted" OR headband* OR temple OR supraorbital  OR ear OR "ear-worn" OR "in-ear" OR earlobe* OR auricular OR concha OR "ear canal"  OR earbud* OR hearable* OR earphone* OR earpiece* OR sternum OR sternal OR chest OR arm OR forearm  )  AND TITLE-ABS-KEY(  (contact W/2 force) OR (contact W/2 pressure) OR (external W/2 pressure) OR (applied W/2 pressure)  OR (applied W/2 force) OR (probe W/2 pressure) OR (probe W/2 force) OR (sensor W/2 pressure)  OR (sensor W/2 force) OR (skin W/2 pressure) OR (interface W/2 pressure) OR (interface W/2 force)  OR (normal W/2 force) OR (normal W/2 pressure) OR (contact W/2 load) OR (loading W/2 force)  OR (contact W/2 stress) OR (wearing W/2 pressure) OR (wearing W/2 force) OR (wearing W/2 tightness)  OR (strap W/2 pressure) OR (strap W/2 force) OR (strap W/2 tightness) OR (strap W/2 tension)  OR (band W/2 tightness) OR (wristband W/2 tightness) OR tightness OR "device fit" OR "wearable fit"  OR "skin contact" OR "sensor contact" OR "probe contact" OR "contact condition*" OR "contact state"  OR "sensor-skin interface" OR "skin-sensor interface" OR "probe-skin interface"  OR "sensor-skin contact" OR "skin-sensor contact" OR "optical coupling" OR "mechanical coupling"  OR "coupling pressure" OR "contact area" OR compression OR "skin compression" OR "tissue compression"  OR "vascular compression" OR "vascular occlusion" OR "transmural pressure" OR "skin indentation"  OR "indentation force"  )  AND TITLE-ABS-KEY(  "signal quality" OR "signal-to-noise" OR SNR OR artifact* OR "motion artifact*" OR noise  OR "waveform morpholog*" OR waveform OR morphology OR "pulse waveform" OR "feature extraction" OR feature*  OR "fiducial point*" OR "foot point" OR "systolic peak" OR "diastolic peak" OR "dicrotic notch"  OR "AC/DC" OR "AC component*" OR "DC component*" OR "perfusion index" OR "pulse amplitude"  OR "pulse area" OR "rise time" OR "reflection index" OR "stiffness index" OR "augmentation index"  OR "pulse arrival time" OR PAT OR "pulse transit time" OR PTT OR "heart rate" OR HR  OR "heart rate variability" OR HRV OR SpO2 OR "oxygen saturation" OR "blood pressure"  OR "blood pressure estimation" OR "blood pressure monitoring" OR "physiological estimation"  OR "estimation accuracy" OR "measurement accuracy" OR calibrat* OR error*  )  )  AND (LIMIT-TO(LANGUAGE, "English")) | 882 |
| **Web of Science Core Collection** | (  TS=(  photoplethysmograph* OR photoplethysmogram* OR photoplethysmographic OR PPG  OR "pulse oximetr*" OR "pulse oximeter*" OR oximeter* OR "optical pulse" OR "pulse wave"  )  AND TS=(  reflectance OR "reflectance mode" OR "reflectance-mode" OR reflective* OR wearable*  OR "wearable device*" OR "wearable sensor*" OR "wearable monitor*" OR "wearable system*"  OR "body-worn" OR "skin-mounted" OR "skin sensor*" OR "wrist-worn" OR wrist OR wristband*  OR watch OR smartwatch* OR "smart watch*" OR ring OR "smart ring*" OR finger* OR fingertip*  OR thumb* OR forehead OR "head-worn" OR "head-mounted" OR headband* OR temple OR supraorbital  OR ear OR "ear-worn" OR "in-ear" OR earlobe* OR auricular OR concha OR "ear canal"  OR earbud* OR hearable* OR earphone* OR earpiece* OR sternum OR sternal OR chest OR arm OR forearm  )  AND TS=(  (contact NEAR/2 force) OR (contact NEAR/2 pressure) OR (external NEAR/2 pressure)  OR (applied NEAR/2 pressure) OR (applied NEAR/2 force) OR (probe NEAR/2 pressure)  OR (probe NEAR/2 force) OR (sensor NEAR/2 pressure) OR (sensor NEAR/2 force)  OR (skin NEAR/2 pressure) OR (interface NEAR/2 pressure) OR (interface NEAR/2 force)  OR (normal NEAR/2 force) OR (normal NEAR/2 pressure) OR (contact NEAR/2 load)  OR (loading NEAR/2 force) OR (contact NEAR/2 stress) OR (wearing NEAR/2 pressure)  OR (wearing NEAR/2 force) OR (wearing NEAR/2 tightness) OR (strap NEAR/2 pressure)  OR (strap NEAR/2 force) OR (strap NEAR/2 tightness) OR (strap NEAR/2 tension)  OR (band NEAR/2 tightness) OR (wristband NEAR/2 tightness) OR tightness OR "device fit"  OR "wearable fit" OR "skin contact" OR "sensor contact" OR "probe contact" OR "contact condition*"  OR "contact state" OR "sensor-skin interface" OR "skin-sensor interface" OR "probe-skin interface"  OR "sensor-skin contact" OR "skin-sensor contact" OR "optical coupling" OR "mechanical coupling"  OR "coupling pressure" OR "contact area" OR compression OR "skin compression" OR "tissue compression"  OR "vascular compression" OR "vascular occlusion" OR "transmural pressure" OR "skin indentation"  OR "indentation force"  )  AND TS=(  "signal quality" OR "signal-to-noise" OR SNR OR artifact* OR "motion artifact*" OR noise  OR "waveform morpholog*" OR waveform OR morphology OR "pulse waveform" OR "feature extraction" OR feature*  OR "fiducial point*" OR "foot point" OR "systolic peak" OR "diastolic peak" OR "dicrotic notch"  OR "AC/DC" OR "AC component*" OR "DC component*" OR "perfusion index" OR "pulse amplitude"  OR "pulse area" OR "rise time" OR "reflection index" OR "stiffness index" OR "augmentation index"  OR "pulse arrival time" OR PAT OR "pulse transit time" OR PTT OR "heart rate" OR HR  OR "heart rate variability" OR HRV OR SpO2 OR "oxygen saturation" OR "blood pressure"  OR "blood pressure estimation" OR "blood pressure monitoring" OR "physiological estimation"  OR "estimation accuracy" OR "measurement accuracy" OR calibrat* OR error*  )  )  AND LA=(English) | 561 |
