# Supplementary material for: Mechanical Contact Conditions in Wearable Reflectance Photoplethysmography: Scoping Review
Source: JMIR Mhealth Uhealth. 2026 Aug 24;14:e99333. doi: 10.2196/99333 (PMC13550977; doi:10.2196/99333)
Supplement: Supplementary file 4 [file mhealth_v14i1e99333_app4.docx]

# Multimedia Appendix 4. Baseline characteristics of the included studies with extracted contact-force/contact-pressure information

|  | **Study** | **Year** | **Type** | **Country or region** | **Study design / focus** | **Sample size and participant characteristics / platform** | **Force/pressure reporting** |
| --- | --- | --- | --- | --- | --- | --- | --- |
|  | He et al [1] | 2026 | Journal | China | · Controlled wrist reflectance PPG study evaluating contact-force effects on signal quality and SpO₂ prediction  · machine-learning models were compared with and without contact-force features. | · CF-controlled dataset: 9 healthy subjects  · WF-PPG dataset: 27 healthy young subjects  · 16 males and 11 females. | · Contact force.  · Unit/range: N  · CF-controlled dataset used 0.2-3.0 N in 0.2 N steps, with ±0.1 N precision.  · Effective contact area: NR. |
| May et al [2] | | 2021 | Journal | United Kingdom | · Static in vitro tissue-vessel phantom study examining how progressively increased sensor contact pressure affects reflectance PPG signal quality and morphological features under simulated blood-pressure states. | · In vitro tissue-vessel phantom  · no human participants  · sex distribution not applicable. | · Contact pressure.  · Unit/range: mmHg  · continuously increased until complete vessel occlusion  · optimal range reported as 35.1-48.1 mmHg.  · Effective contact area: not reported. |
|  | Sim et al [3] | 2018 | Journal | South Korea | · Contact-force-regulated wrist PPG platform study using active closed-loop force control to evaluate force-related waveform stability and posture-related variability. | · Healthy human-subject validation  · main posture-change experiment: 1 healthy subject  · additional contact-force dependency experiment: 4 subjects. Male female distribution and health status: NR. | · Contact force.  · Unit/range: N  · target force 0.6 N  · measurable range 0.2-4.0 N.  · Effective contact area: not reported. |
|  | Chandrasekhar et al [4] | 2020 | Journal | United States | · Contact-pressure experiment evaluating whether PPG-derived PAT is affected by PPG sensor contact pressure  · the study focused on potential cuffless BP estimation using PPG timing features. | · 17 healthy subjects in the main experiment  · male/female distribution: NR in the summarized table. | · Contact pressure.  · Unit/range: pressure varied over physiologically relevant levels  · exact range not specified in the summarized table.  · Effective contact area: NR. |
|  | Dresher and Mendelson [5] | 2006 | Conference | United States | · Forehead reflectance pulse-oximetry walking study under different contact pressures  · oxygen-saturation monitoring context. | · 10 healthy volunteers  · male/female distribution: NR. | · Contact pressure was investigated under different pressure conditions. Detailed unit range was not reported in the summarized table. |
|  | Sirkiä et al [6] | 2021 | Conference | Finland | · Quasi-static in vivo finger multi-wavelength PPG pressure-ramp study examining how controlled external finger pressure affects wavelength-dependent PPG waveforms, vascular occlusion/opening behavior, and pulse-foot timing. | · 3 volunteers  · 1 female  · health status and age: NR. | · External pressure/contact pressure.  · Unit/range: mmHg  · pressure was increased above systolic blood pressure and then released.  · Geometry/contact area: MWPPG sensor PCB diameter approximately 9.6 mm  · effective skin-contact area not reported. |
|  | Scardulla et al [7] | 2025 | Journal | Italy | · Dynamic treadmill wrist PPG study evaluating the combined influence of contact pressure and activity level on HR measurement performance. | · 25 healthy volunteer subjects  · male/female distribution: NR. | · Contact pressure.  · Unit/range: mmHg  · 20, 60, and 75 mmHg.  · Effective contact area: NR. |
|  | Liu et al [8] | 2024 | Journal | China, Hong Kong | · Wearable multi-channel PPG array study emphasizing synchronous spatial monitoring of local contact-force variation and its association with PPG waveform behavior. | · Sample size and participant health status: NR. | · Contact-force-related signal.  · Unit/range: not reported.  · Geometry/contact area: wrist-contact PPG and piezoelectric sensors  · sensor diameter reported as 12 mm, but effective skin-contact area for pressure conversion was not reported. |
|  | Přibil et al [9] | 2025 | Journal | Slovakia | · Wearable PPG optical sensor study integrating contact-pressure-force, skin temperature, and humidity measurement to evaluate PPG changes under five force levels. | · 6 healthy volunteers: 4 males and 2 females  · age 56 ± 8 years  · healthy volunteers. | · Contact force  · authors used the term CPF.  · Unit/range: N  · five force levels. Male: 0.147-1.078 N  · female: 0.147-0.753 N.  · FSR active sensing area was 4 mm in diameter, but effective skin-contact area was not reported  · pressure in mmHg kPa not reported. |
|  | Fortin et al [10] | 2020 | Conference | Canada | · Contact-force estimation study using raw PPG features and a bagged-tree model, with ground-truth force recorded during development. | · 1 participant: male, 28 years old, right-handed, pale Caucasian skin tone  · self-administered protocol because of COVID-19 self-isolation restrictions. | · Contact force.  · Unit/range: N  · 0-2.6 N in 0.2 N increments.  · Effective contact area: not reported. |
|  | Charlton et al [11] | 2025 | Journal | United Kingdom | · Large-scale wrist reflectance PPG signal-quality study examining how posture, sensor height relative to the heart, and skin-contact state influence SNR, perfusion index, and template-matching quality. | · 1,142 participants from the Aurora-BP dataset with varying ages and health statuses. | · Contact force/pressure was not directly manipulated.  · Unit/range: not reported.  · Effective contact area: not reported. |
|  | Lambert Cause et al [12] | 2024 | Journal | Spain | · In vivo fingertip PPG study examining how different normalized contact-force levels affect multiple signal-quality indices and reflect different aspects of PPG signal quality. | · 11 healthy participants  · male/female distribution: NR. | · Contact force.  · Unit/range: normalized unit  · CF normalized from 0 to 1 and divided into five bins.  · Effective contact area: not reported. |
|  | Sirkiä et al [13] | 2024 | Journal | Finland | · In vivo finger multi-wavelength PPG pressure protocol examining pressure-dependent AC/DC responses, feature stability, and SpO₂-related behavior. | · 19 healthy participants with multi-wavelength finger PPG  · male/female distribution: NR. | · Contact pressure/external finger-cuff pressure.  · Unit/range: mmHg  · finger-cuff pressure applied in pressure steps, including 10 mmHg increments in the pressure-step analysis.  · Effective skin-contact area: not reported. |
|  | Ho et al [14] | 2025 | Journal | United States | · Static/quasi-static seated wrist-finger dual-channel PPG dataset study examining wrist contact-pressure-related morphology changes, with fingertip PPG recorded simultaneously as a reference. | · 27 healthy young participants  · 16 males and 11 females  · age 24.3 ± 2.74 years. | · Contact pressure.  · Unit/range: load-cell reading  · varied or gradually increased across six seated sessions.  · Geometry/contact area: load cell placed between the wrist PPG sensor and movable jaw  · effective skin-contact area not reported. |
|  | Chan et al [15] | 2021 | Journal | United States | · Wearable reflectance pulse-oximetry study at the sternum  · continuous SpO₂ monitoring context. | · Total sample size, sex distribution, and participant characteristics: NR. | · Contact force/pressure was not reported as a quantified variable in the summarized table. |
|  | Scardulla et al [16] | 2020 | Journal | Italy | · Dynamic step-exercise wrist PPG study evaluating HR measurement under different contact pressures and activity rates. | · 17 subjects  · 12 males and 5 females  · age 36 ± 11 years  · health status: NR. | · Contact pressure.  · Unit/range: mmHg  · 12, 33, and 54 mmHg.  · Geometry/contact area: contact area = 473 mm². |
|  | Hu et al [17] | 2025 | Conference | United States | · Morphology-aware wrist PPG HRV estimation study under sedentary scenarios  · the model used raw PPG waveform information and pressure-induced morphology labels to estimate NN intervals and beat count for HRV computation. | · Public WF-PPG dataset  · 27 healthy young subjects  · 16 males and 11 females. | · Pressure-induced morphology label, not direct pressure intervention.  · Unit/range: not applicable as a direct pressure-level experiment.  · Effective contact area: NR. |
|  | Dresher and Mendelson [18] | 2006 | Conference | United States | · In vivo forehead reflectance pulse-oximeter housing study evaluating whether a two-part housing design can reduce contact-pressure effects  · SpO₂ and HR readings were compared between a control housing and two new housing designs under stepwise contact-pressure loading. | · 10 healthy volunteers  · age 19-24 years  · male/female distribution: NR. | · Contact pressure.  · Unit/range: mmHg  · 0-399, 400-699, 700-999, and 1000-1200 mmHg.  · Effective contact area: NR. |
|  | Grabovskis et al [19] | 2013 | Journal | Latvia | · In vivo conduit-artery PPG study examining how probe contact pressure affects morphology-derived arterial-stiffness assessment. | · 5 healthy subjects  · male/female distribution: NR. | · Contact pressure.  · Unit/range: kPa  · probe CP varied from 0 to 15 kPa.  · Effective contact area: not reported. |
|  | Teng and Zhang [20] | 2007 | Journal | China, Hong Kong | · Combined theoretical modeling and human-subject experimental validation study examining nonlinear contact-force–related changes in finger PPG-derived PTT through the pressure–volume relationship of the finger arterial wall. | · 30 young healthy participants: 20 males and 10 females; aged 20–29 years.  · 6 older healthy participants: 2 males and 4 females; aged 44–53 years.  · An additional repeatability experiment involved 10 young healthy participants: 6 males and 4 females. | · Contact force.  · Unit/range: N.  · Twelve discrete contact-force levels from 0.1 to 1.8 N were applied in random order at the right index fingertip.  · Effective contact area: not reported. |
|  | Fine and Kaminsky [21] | 2019 | Journal | Israel | · Reflection pulse-oximetry study examining how applied pressure affects the dual-wavelength PPG-derived Gamma parameter used in SpO₂ calculation. | · Experimental reflection pulse-oximetry measurements  · sample size and participant characteristics: NR. | · Applied pressure/local pressure.  · Unit/range: pressure controlled using inflatable silicone cushions  · pressure up to 120 torr was used in the pressure-increase/decrease experiment.  · Effective contact area: not reported. |

For studies appearing in multiple evidence tables, the row summarizes the most relevant study-design/focus, participant/platform, and force/pressure-reporting information available in the current manuscript tables. NR = not reported; PPG = photoplethysmography; SpO₂ = peripheral oxygen saturation; HRV = heart-rate variability; PAT = pulse arrival time; PTT = pulse transit time; BP = blood pressure; CF = contact force.

**References**

1. He Z, Cai Z, Sun J, Li J, Liu C, Yang C. A preliminary quantitative analysis of contact force effects on peripheral oxygen saturation prediction from reflectance photoplethysmography. IEEE Sens J. 2026;PP(99):1-1. doi:10.1109/JSEN.2026.3671734
2. May JM, Mejia-Mejia E, Nomoni M, Budidha K, Choi C, Kyriacou PA. Effects of contact pressure in reflectance photoplethysmography in an in vitro tissue-vessel phantom. Sensors (Basel). 2021;21(24):8421. doi:10.3390/s21248421
3. Sim JK, Ahn B, Doh I. A contact-force regulated photoplethysmography (PPG) platform. AIP Adv. 2018;8(4):045210. doi:10.1063/1.5020914
4. Chandrasekhar A, Yavarimanesh M, Natarajan K, Hahn JO, Mukkamala R. PPG sensor contact pressure should be taken into account for cuff-less blood pressure measurement. IEEE Trans Biomed Eng. 2020;67(11):3134-3140. doi:10.1109/TBME.2020.2976989
5. Dresher RP, Mendelson Y. Reflectance forehead pulse oximetry: effects of contact pressure during walking. Conf Proc IEEE Eng Med Biol Soc. 2006;2006:3529-3532. doi:10.1109/IEMBS.2006.260136
6. Sirkia JP, Panula T, Kaisti M. The effects of external pressure on multi-wavelength photoplethysmography signals. Comput Cardiol. 2021;48:1-4. doi:10.23919/CinC53138.2021.9662771
7. Scardulla F, Cosoli G, Gnoffo C, Antognoli L, Bongiorno F, Diana G, Scalise L, D'Acquisto L, Arnesano M. Experimental analysis on the effect of contact pressure and activity level as influencing factors in PPG sensor performance. Sensors (Basel). 2025;25(14):4477. doi:10.3390/s25144477
8. Liu ZJ, Xiang T, Zhou RS, Ji N, Zhang YT. A multi-channel photoplethysmography array with contact-force regulation for tonoarteriographic imaging. Conn Health Telemed. 2024;3:300001. doi:10.20517/chatmed.2023.11
9. Pribil J, Pribilova A, Dermek T. Wearable PPG multi-sensor for skin humidity, temperature, and contact pressure measurement in weak magnetic field environment: first-step experiments. Bioengineering (Basel). 2025;12(12):1361. doi:10.3390/bioengineering12121361
10. Fortin PE, Blum JR, Weill-Duflos A, Cooperstock JR. Contact force estimation from raw photoplethysmogram signal. In: 2020 IEEE Sensors. IEEE; 2020:1-4. doi:10.1109/SENSORS47125.2020.9278658
11. Charlton PH, Marozas V, Mejia-Mejia E, Kyriacou PA, Mant J. Determinants of photoplethysmography signal quality at the wrist. PLOS Digit Health. 2025;4(6):e0000585. doi:10.1371/journal.pdig.0000585
12. Lambert Cause J, Sole Morillo A, Garcia-Naranjo JC, Stiens J, da Silva B. The impact of contact force on signal quality indices in photoplethysmography measurements. Applied Sciences. 2024;14(13):5704. doi:10.3390/app14135704
13. Sirkia JP, Panula T, Kaisti M. Investigating the impact of contact pressure on photoplethysmograms. Biomed Eng Adv. 2024;7:100123. doi:10.1016/j.bea.2024.100123
14. Ho MY, Pham HM, Saeed A, Ma D. WF-PPG: a wrist-finger dual-channel dataset for studying the impact of contact pressure on PPG morphology. Sci Data. 2025;12(1):200. doi:10.1038/s41597-025-04453-7
15. Chan M, Ganti VG, Heller JA, Abdallah CA, Etemadi M, Inan OT. Enabling continuous wearable reflectance pulse oximetry at the sternum. Biosensors (Basel). 2021;11(12):521. doi:10.3390/bios11120521
16. Scardulla F, D'Acquisto L, Colombarini R, Hu S, Pasta S, Bellavia D. A study on the effect of contact pressure during physical activity on photoplethysmographic heart rate measurements. Sensors (Basel). 2020;20(18):5052. doi:10.3390/s20185052
17. Hu C, Pham HM, Ma D. Morphology-aware HRV estimation from wrist PPG in sedentary scenarios. In: Adjunct Proceedings of the 2025 ACM International Joint Conference on Pervasive and Ubiquitous Computing and the 2025 ACM International Symposium on Wearable Computers. Association for Computing Machinery; 2025:745-750. doi:10.1145/3714394.3756171
18. Dresher RP, Mendelson Y. A new reflectance pulse oximeter housing to reduce contact pressure effects. In: Proceedings of the IEEE 32nd Annual Northeast Bioengineering Conference. IEEE; 2006:35-36.
19. Grabovskis A, Marcinkevics Z, Rubins U, Kviesis-Kipge E. Effect of probe contact pressure on the photoplethysmographic assessment of conduit artery stiffness. J Biomed Opt. 2013;18(2):027004. doi:10.1117/1.JBO.18.2.027004
20. Teng XF, Zhang YT. Theoretical study on the effect of sensor contact force on pulse transit time. IEEE Trans Biomed Eng. 2007;54(8):1490-1498. doi:10.1109/TBME.2007.900815
21. Fine I, Kaminsky A. Possible error in reflection pulse oximeter readings as a result of applied pressure. J Healthc Eng. 2019;2019:7293813. doi:10.1155/2019/7293813
