# Supplementary material for: Mechanical Contact Conditions in Wearable Reflectance Photoplethysmography: Scoping Review
Source: JMIR Mhealth Uhealth. 2026 Aug 24;14:e99333. doi: 10.2196/99333 (PMC13550977; doi:10.2196/99333)
Supplement: Supplementary file 5 [file mhealth_v14i1e99333_app5.docx]

**Multimedia Appendix 5. Supporting Evidence Tables for Contact-Condition–Related Findings in Wearable Reflectance Photoplethysmography**

Table A1. Reported signal and signal-quality responses under different contact conditions.

| **Study** | **Study design / application context** | **Sample size and participant characteristics / platform** | **Body site** | **Force/pressure reporting** | **Metric(s)** | **Main finding** |
| --- | --- | --- | --- | --- | --- | --- |
| Lambert Cause et al [1] | In vivo fingertip PPG study examining how different normalized contact-force levels relate to multiple signal-quality indices. | • 11 healthy participants. • Male/female distribution: NR. | Fingertip. | • Contact force. • Normalized unit; CF normalized from 0 to 1 and divided into five bins. • Effective contact area: not reported. | SSQI, KSQI, RSQI, ZSQI, ESQI, PSQI. | Different SQIs favored different force ranges: • 0.2–0.4 was more favorable for perfusion/blood-flow information. • 0.4–0.6 better preserved complex waveform detail and physiological representativeness. |
| May et al [2] | Static in vitro tissue-vessel phantom study examining progressively increased contact pressure in relation to reflectance PPG SNR and waveform morphology. | • Tissue-vessel phantom. • No human participants. | Above the embedded vessel in the phantom. | • Contact pressure. • Unit/range: mmHg; progressively increased until complete vessel occlusion. • Effective contact area: not reported. | SNR. | • SNR increased and then decreased with contact pressure. • After complete vessel occlusion, SNR could become negative. |
| He et al [3] | Force-controlled wrist reflectance PPG study evaluating contact-force–related differences in high-frequency noise, baseline drift, motion artifact, and morphology-related signal quality for SpO₂ prediction. | • 9 healthy participants in the force-controlled dataset. • 27 healthy young participants in WF-PPG; 16 males and 11 females. | Wrist. | • Contact force. • Unit/range: N; 0.2–3.0 N in 0.2 N steps in the force-controlled dataset. • Effective contact area: not reported. | P index for high-frequency noise; Q index for baseline drift; R index for motion artifact. | • P decreased continuously as force increased. • Q remained relatively stable. • R improved and then plateaued at higher force. • Moderate/heavy contact produced a steadier baseline and clearer morphology than loose contact. |

Table A2. Reported waveform-morphology and feature responses across contact conditions.

| **Study** | **Study design / waveform-feature focus** | **Sample size and participant characteristics / platform** | **Measurement site** | **Force/pressure reporting** | **Evaluation level** | **Affected signal/feature dimension** | **Main result** |
| --- | --- | --- | --- | --- | --- | --- | --- |
| May et al [2] | Static in vitro tissue-vessel phantom study examining contact-pressure–related PPG amplitude and SNR responses. | • Tissue-vessel phantom. • No human participants. | Above the embedded vessel in the phantom. | • Contact pressure. • Unit/range: mmHg; progressively increased until vessel restriction/occlusion. • Effective contact area: not reported. | Waveform level. | Amplitude / SNR. | PPG amplitude and SNR increased initially with pressure and decreased when pressure became excessive and restricted the vessel. |
| Ho et al [4] | Static/quasi-static seated wrist-finger dual-channel PPG dataset study examining wrist contact-pressure–related morphology differences. | • 27 healthy young participants. • 16 males and 11 females; age 24.3 ± 2.74 years. | Wrist PPG; fingertip PPG used as reference. | • Contact pressure. • Unit/range: load-cell reading; varied across six seated sessions. • Effective skin-contact area: not reported. | Waveform level. | Wrist PPG waveform morphology. | Waveforms evolved from low-force single-peaked shapes to clearer double-peaked morphologies at suitable force and then back toward distorted single-peaked forms at higher force. |
| Sirkiä et al [5] | In vivo finger multi-wavelength PPG pressure protocol examining pressure-dependent AC/DC and wavelength responses. | • 19 healthy participants. • Male/female distribution: NR. | Palmar side of the left index fingertip. | • Contact pressure/external finger-cuff pressure. • Unit/range: mmHg; pressure applied in steps. • Effective skin-contact area: not reported. | Waveform level. | Baseline / DC component. | Contact pressure was associated with differences in both AC and DC components, and the response varied across wavelengths. |
| Sirkiä et al [5] | In vivo finger multi-wavelength PPG pressure protocol examining feature stability across pressure levels. | • 19 healthy participants. • Male/female distribution: NR. | Palmar side of the left index fingertip. | • Contact pressure/external finger-cuff pressure. • Unit/range: mmHg; pressure applied in steps. • Effective skin-contact area: not reported. | Feature level. | Overall feature response. | Multiple features varied across pressure levels but not equally; many were more stable under slight pressure or near diastolic pressure. |
| May et al [2] | Static in vitro tissue-vessel phantom feature-stability study under progressively increased contact pressure. | • Tissue-vessel phantom. • No human participants. | Above the embedded vessel in the phantom. | • Contact pressure. • Unit/range: mmHg; progressively increased until vessel occlusion. • Effective contact area: not reported. | Feature level. | Time-domain and amplitude-related features. | Cycle length, pulse width, and rise/fall times changed less across pressure levels, whereas pulse amplitude and area showed larger differences. |
| Grabovskis et al [6] | In vivo conduit-artery PPG study examining probe-contact-pressure–related differences in morphology-derived arterial-stiffness assessment. | • 5 healthy subjects. • Male/female distribution: NR. | Posterior tibial, femoral, and popliteal arteries. | • Contact pressure. • Unit/range: kPa; 0–15 kPa. • Effective contact area: not reported. | Feature level. | Second-derivative PPG peak ratio b/a. | The AC PPG second-derivative b/a ratio differed across probe pressures, indicating pressure sensitivity of this morphology-derived index. |
| Fine and Kaminsky [7] | Reflection pulse-oximetry study examining applied-pressure–related differences in the dual-wavelength Gamma parameter used in SpO₂ calculation. | • Experimental reflection pulse-oximetry measurements. • Sample size and participant characteristics: NR. | Fingertip, wrist, and forehead. | • Applied/local pressure. • Pressure controlled using inflatable silicone cushions; up to 120 torr. • Effective contact area: not reported. | Feature level. | Dual-wavelength ratio parameter Gamma. | Gamma increased with applied pressure, indicating differences in the red/infrared PPG relationship used for oxygen-saturation calculation. |
| Sirkiä et al [5]; May et al [2] | Combined evidence from in vivo MWPPG and in vitro phantom protocols on pressure-sensitive morphology-related features. | • Sirkiä et al: 19 healthy participants. • May et al: tissue-vessel phantom. | Fingertip; above the embedded vessel in the phantom. | • Finger-cuff external pressure and direct contact pressure. • Unit/range: mmHg. • Effective skin-contact area: not reported. | Feature level. | Morphological features. | Geometric features reflected both amplitude and contour differences and were comparatively pressure-sensitive in the tested protocols. |

Table A3. Reported artifact susceptibility and temporal stability across contact conditions.

| **Study** | **Study design / artifact-tracking focus** | **Sample size and participant characteristics** | **Measurement site** | **Force/pressure reporting** | **Most susceptible target** | **Main observation and finding** | **Suggested/observed range** |
| --- | --- | --- | --- | --- | --- | --- | --- |
| Sim et al [8] | Wrist posture-change experiment using a contact-force-regulated PPG platform to evaluate force-related artifact susceptibility and temporal stability. | • 4 participants in the contact-force dependency experiment. • Sex distribution and health status: NR. | Radial artery at the wrist. | • Contact force. • Unit/range: N; target 0.6 N; measurable range 0.2–4.0 N. • Effective contact area: not reported. | PPGA amplitude was more force-sensitive than PPGI interval. | In the lower or unstable contact-force range, posture-induced variation mainly involved PPGA, whereas PPGI was less sensitive. Force stabilization reduced posture-related waveform variability. | 0.6 N; amplitude-dominated indices may require tighter force control. |
| Sirkiä et al [9] | Quasi-static multi-wavelength finger pressure-ramp experiment examining wavelength-dependent artifact susceptibility and timing stability. | • 3 volunteers; 1 female. • Health status and age: NR. | Right index fingertip. | • External/contact pressure. • Unit/range: mmHg; increased above systolic BP and then released. • Sensor PCB diameter ≈9.6 mm; effective skin-contact area not reported. | Blue and green channels were more pressure-sensitive than red and infrared channels. | Short-wavelength channels showed earlier occlusion-like behavior, whereas red/infrared channels preserved more stable pulse-foot timing. | Red/infrared channels were more robust under varying pressure. |
| Scardulla et al [10] | Dynamic step-exercise wrist PPG protocol examining pressure-related artifact susceptibility during movement. | • 17 subjects; 12 males and 5 females. • Age 36 ± 11 years. | Wrist. | • Contact pressure. • Unit/range: mmHg; 12, 33, and 54 mmHg. • Contact area: 473 mm². | Low-pressure wrist PPG HR tracking, especially at 12 mmHg. | At 12 mmHg, poor contact and motion artifacts reduced PPG-HR agreement with ECG; 54 mmHg provided the best group-level dynamic tracking among the tested pressures. | 54 mmHg at group level, with individual variation. |
| Scardulla et al [11] | Dynamic treadmill wrist PPG protocol examining HR tracking across contact pressures and activity levels. | • 25 healthy volunteers. • Male/female distribution: NR. | Wrist. | • Contact pressure. • Unit/range: mmHg; 20, 60, and 75 mmHg. • Effective contact area: not reported. | Dynamic wrist PPG waveform quality and HR tracking at low and high pressure extremes. | At 20 mmHg, insufficient contact and motion artifacts predominated; at 75 mmHg, higher activity reduced pulsatile oscillation; 60 mmHg produced the best overall group performance. | 60 mmHg at group level; individualized optimization performed better. |

Table A4. Reported downstream estimation outcomes across contact conditions.

| **Study** | **Study design / downstream-estimation focus** | **Sample size and participant characteristics / platform** | **Measurement site / reference** | **Force/pressure reporting** | **Downstream task / outcome** | **Main finding** |
| --- | --- | --- | --- | --- | --- | --- |
| He et al [3] | Controlled wrist reflectance PPG study evaluating contact-force–related signal-quality responses and SpO₂ prediction; models with and without force features were compared. | • CF-controlled dataset: 9 healthy subjects. • WF-PPG: 27 healthy young subjects; 16 males and 11 females. | Wrist reflectance PPG; fingertip SpO₂ used as reference. | • Contact force. • Unit/range: N; 0.2–3.0 N in 0.2 N steps, ±0.1 N precision. • Effective contact area: NR. | SpO₂ estimation. | Adding contact-force features improved prediction compared with PPG-only and equation-based models; the CF + PPG model achieved an MAE of 0.8811% in WF-PPG evaluation. |
| Chandrasekhar et al [12] | Contact-pressure experiment evaluating PPG-derived PAT for potential cuffless BP estimation. | • 17 healthy subjects in the main experiment. • Male/female distribution: NR. | Finger PPG; ECG used to compute PAT; finger-cuff BP used for BP-related comparison. | • Contact pressure varied over physiologically relevant levels. • Exact range not specified in the summarized table. • Effective contact area: NR. | PAT-based BP-related assessment. | Contact pressure shifted PPG fiducial-point timing. PATfoot and PATpeak shifts corresponded to approximately 11 and 20 mmHg of BP estimation error, respectively. |
| Teng and Zhang [13] | Combined theoretical modeling and human-subject experimental validation examining nonlinear contact-force–related differences in PPG-derived PTT. | • 30 young healthy subjects (20 males, 10 females; 20–29 years). • 6 older healthy subjects (2 males, 4 females; 44–53 years). • Repeatability experiment in 10 young subjects. | Right index fingertip PPG; ECG lead I R wave used as the proximal timing reference. | • Contact force. • 12 discrete levels from 0.1 to 1.8 N, applied in random order. • Effective contact area: not reported. | PTT. | PTT increased as contact force approached approximately zero transmural pressure and then remained near a constant level. Experimental results were consistent with the theoretical pattern. |
| Dresher and Mendelson [14] | Forehead reflectance pulse-oximeter housing study evaluating whether a two-part housing design reduces contact-pressure–related measurement variation. | • 10 healthy volunteers. • Age 19–24 years. • Male/female distribution: NR. | Forehead sensor ≈3 cm above eyebrow; fingertip Nonin sensor as independent reference. | • Contact pressure. • Unit/range: mmHg; 0–399, 400–699, 700–999, and 1000–1200 mmHg. • Effective contact area: NR. | SpO₂ and HR estimation. | Large pressure variation was associated with differences in forehead PPG amplitude and SpO₂/HR accuracy. Two-part housings reduced error and variability compared with the control housing. |
| Scardulla et al [10] | Dynamic step-exercise wrist PPG study evaluating HR measurement at different contact pressures and activity rates. | • 17 subjects; 12 males and 5 females. • Age 36 ± 11 years. | Wrist PPG; ECG chest strap as reference. | • Contact pressure. • Unit/range: 12, 33, and 54 mmHg. • Contact area: 473 mm². | HR estimation during physical activity. | Contact pressure was associated with differences in PPG-HR agreement with ECG; 54 mmHg showed the best group-level performance among the tested conditions. |
| Scardulla et al [11] | Dynamic treadmill wrist PPG study evaluating contact pressure and activity level in relation to HR performance. | • 25 healthy volunteers. • Male/female distribution: NR. | Wrist PPG prototype; ECG-based device as reference. | • Contact pressure. • Unit/range: 20, 60, and 75 mmHg. • Effective contact area: NR. | HR estimation across activity levels. | Contact pressure and activity level were associated with differences in accuracy and precision. The 60-mmHg condition gave the best group-level performance, and individualized pressure further reduced uncertainty. |
| Hu et al [15] | Morphology-aware wrist PPG HRV estimation using raw waveforms and pressure-induced morphology labels to estimate NN intervals and beat count. | • Public WF-PPG dataset. • 27 healthy young subjects; 16 males and 11 females. | Wrist PPG; fingertip PPG/reference measurements used for comparison. | • Pressure-induced morphology label rather than direct pressure intervention. • Physical pressure unit not applicable. • Effective contact area: NR. | HRV estimation. | Morphology-aware modeling improved HRV-related estimation compared with pressure-agnostic baselines and supported estimation of NN intervals and beat count. |

**Table A5. Reported nonlinear response patterns and study-specific force/pressure ranges.**

| **Study** | **Study design / nonlinear-response focus** | **Sample size and participant characteristics / platform** | **Site** | **Force/pressure reporting** | **Task / metric** | **Nonlinear behavior** | **Conclusion / preferred range** |
| --- | --- | --- | --- | --- | --- | --- | --- |
| May et al [2] | Static phantom study examining nonlinear contact-pressure responses in PPG amplitude and SNR. | • Tissue-vessel phantom. • No human participants. | Above the embedded vessel in the phantom. | • Contact pressure. • Unit/range: mmHg; progressively increased until complete vessel occlusion. • Effective contact area: not reported. | SNR; waveform amplitude. | Amplitude and SNR increased with pressure, peaked, and then declined; after full occlusion, SNR could become negative. | Peak SNR occurred at approximately 35.1–48.2 mmHg in this phantom protocol. |
| May et al [2] | Static phantom feature-response study comparing pressure sensitivity across 17 PPG morphology features. | • Tissue-vessel phantom. • No human participants. | Above the embedded vessel in the phantom. | • Contact pressure. • Unit/range: mmHg; progressively increased until occlusion. • Effective contact area: not reported. | 17 morphological features. | Time-related features changed less, whereas amplitude and geometric features changed more across pressure levels. | Time-related features were comparatively stable; favorable pressure depended on the feature and task. |
| Lambert Cause et al [1] | In vivo fingertip study examining nonlinear normalized-contact-force patterns in perfusion-related signal quality. | • 11 healthy participants. • Male/female distribution: NR. | Index fingertips of both hands. | • Contact force. • Normalized 0–1 and divided into five bins. • Effective contact area: not reported. | PSQI / perfusion index. | PI was higher in low-to-moderate force ranges and declined at higher force. | 0.2–0.4 was more suitable for perfusion- and blood-flow-related information in the tested protocol. |
| Lambert Cause et al [1] | In vivo fingertip study examining nonlinear normalized-contact-force patterns in waveform-quality indices. | • 11 healthy participants. • Male/female distribution: NR. | Index fingertips of both hands. | • Contact force. • Normalized 0–1 and divided into five bins. • Effective contact area: not reported. | SSQI, KSQI, RSQI, ESQI, ZSQI. | Most SQIs were less favorable at both low- and high-force extremes; waveform entropy decreased and distortion increased at high force. | 0.4–0.6 better preserved complex waveform detail and overall signal quality in the tested protocol. |
| Sirkiä et al [5] | In vivo finger MWPPG pressure-step study examining pressure-related differences in SpO₂ estimation. | • 19 healthy participants. • Male/female distribution: NR. | Palmar side of the left index fingertip. | • Contact/external finger-cuff pressure. • Unit/range: mmHg; 10-mmHg increments. • Effective skin-contact area: not reported. | SpO₂. | SpO₂ was relatively stable at low pressure and increased as pressure approached diastolic and mean arterial pressure. | A lower stable-pressure range was more reliable; high pressure was not favorable. |
| Sirkiä et al [5] | In vivo finger MWPPG study examining nonlinear feature responses and PAT stability. | • 19 healthy participants. • Male/female distribution: NR. | Palmar side of the left index fingertip. | • Contact/external finger-cuff pressure. • Unit/range: mmHg; 10-mmHg increments. • Effective skin-contact area: not reported. | PPG features; PAT. | Amplitude and PI followed an inverted-U pattern; PATfoot was more stable than PATpeak. | For many features, approximately 20–30 mmHg up to diastolic pressure was preferable; the favorable range depended on the task. |
| Teng and Zhang [13] | Combined theoretical modeling and human-subject experimental validation examining nonlinear sensor-contact-force responses in PPG-derived PTT. | • 30 young healthy subjects and 6 older healthy subjects. • Repeatability experiment in 10 young subjects. | Right index fingertip. | • Contact force. • Unit/range: N; 12 discrete levels from 0.1 to 1.8 N in random order. • Effective contact area: not reported. | PTT. | PTT increased with contact force under positive transmural pressure, reached a maximum near zero transmural pressure, and then remained nearly constant under negative transmural pressure; the experimental pattern agreed with the model. | Contact force should be controlled during PTT measurement; no universal preferred force range was established. |

Abbreviations: BP = blood pressure; CF = contact force; ECG = electrocardiography; HR = heart rate; HRV = heart-rate variability; MAE = mean absolute error; NR = not reported; PAT = pulse arrival time; PPG = photoplethysmography; PTT = pulse transit time; SNR = signal-to-noise ratio; SpO₂ = peripheral oxygen saturation.

**References**

1. Lambert Cause J, Sole Morillo A, Garcia-Naranjo JC, Stiens J, da Silva B. The impact of contact force on signal quality indices in photoplethysmography measurements. Applied Sciences. 2024;14(13):5704. doi:10.3390/app14135704
2. May JM, Mejia-Mejia E, Nomoni M, Budidha K, Choi C, Kyriacou PA. Effects of contact pressure in reflectance photoplethysmography in an in vitro tissue-vessel phantom. Sensors (Basel). 2021;21(24):8421. doi:10.3390/s21248421
3. He Z, Cai Z, Sun J, Li J, Liu C, Yang C. A preliminary quantitative analysis of contact force effects on peripheral oxygen saturation prediction from reflectance photoplethysmography. IEEE Sens J. 2026;PP(99):1-1. doi:10.1109/JSEN.2026.3671734
4. Ho MY, Pham HM, Saeed A, Ma D. WF-PPG: a wrist-finger dual-channel dataset for studying the impact of contact pressure on PPG morphology. Sci Data. 2025;12(1):200. doi:10.1038/s41597-025-04453-7
5. Sirkia JP, Panula T, Kaisti M. Investigating the impact of contact pressure on photoplethysmograms. Biomed Eng Adv. 2024;7:100123. doi:10.1016/j.bea.2024.100123
6. Grabovskis A, Marcinkevics Z, Rubins U, Kviesis-Kipge E. Effect of probe contact pressure on the photoplethysmographic assessment of conduit artery stiffness. J Biomed Opt. 2013;18(2):027004. doi:10.1117/1.JBO.18.2.027004
7. Fine I, Kaminsky A. Possible error in reflection pulse oximeter readings as a result of applied pressure. J Healthc Eng. 2019;2019:7293813. doi:10.1155/2019/7293813
8. Sim JK, Ahn B, Doh I. A contact-force regulated photoplethysmography (PPG) platform. AIP Adv. 2018;8(4):045210. doi:10.1063/1.5020914
9. Sirkia JP, Panula T, Kaisti M. The effects of external pressure on multi-wavelength photoplethysmography signals. Comput Cardiol. 2021;48:1-4. doi:10.23919/CinC53138.2021.9662771
10. Scardulla F, D'Acquisto L, Colombarini R, Hu S, Pasta S, Bellavia D. A study on the effect of contact pressure during physical activity on photoplethysmographic heart rate measurements. Sensors (Basel). 2020;20(18):5052. doi:10.3390/s20185052
11. Scardulla F, Cosoli G, Gnoffo C, Antognoli L, Bongiorno F, Diana G, Scalise L, D'Acquisto L, Arnesano M. Experimental analysis on the effect of contact pressure and activity level as influencing factors in PPG sensor performance. Sensors (Basel). 2025;25(14):4477. doi:10.3390/s25144477
12. Chandrasekhar A, Yavarimanesh M, Natarajan K, Hahn JO, Mukkamala R. PPG sensor contact pressure should be taken into account for cuff-less blood pressure measurement. IEEE Trans Biomed Eng. 2020;67(11):3134-3140. doi:10.1109/TBME.2020.2976989
13. Teng XF, Zhang YT. Theoretical study on the effect of sensor contact force on pulse transit time. IEEE Trans Biomed Eng. 2007;54(8):1490-1498. doi:10.1109/TBME.2007.900815
14. Dresher RP, Mendelson Y. A new reflectance pulse oximeter housing to reduce contact pressure effects. In: Proceedings of the IEEE 32nd Annual Northeast Bioengineering Conference. IEEE; 2006:35-36. doi:10.1109/NEBC.2006.1629746
15. Hu C, Pham HM, Ma D. Morphology-aware HRV estimation from wrist PPG in sedentary scenarios. In: Adjunct Proceedings of the 2025 ACM International Joint Conference on Pervasive and Ubiquitous Computing and the 2025 ACM International Symposium on Wearable Computers. Association for Computing Machinery; 2025:745-750. doi:10.1145/3714394.3756171
